# Spatiotemporal characterization of water diffusion anomalies in saline solutions using machine learning force field

**DOI:** 10.1126/sciadv.adp9662

**Published:** 2024-12-11

**Authors:** Ji Woong Yu, Sebin Kim, Jae Hyun Ryu, Won Bo Lee, Tae Jun Yoon

**Affiliations:** ^1^ Center for AI and Natural Sciences, Korea Institute for Advanced Study, Seoul 02455, Republic of Korea.; ^2^ School of Chemical and Biological Engineering, Institute of Chemical Processes, Seoul National University, Seoul 08826, Republic of Korea.; ^3^ School of Transdisciplinary Innovations, Seoul National University, Seoul 08826, Republic of Korea.

## Abstract

Understanding water behavior in salt solutions remains a notable challenge in computational chemistry. Conventional force fields have shown limitations in accurately representing water’s properties across different salt types (chaotropes and kosmotropes) and concentrations, demonstrating the need for better methods. Machine learning force field applications in computational chemistry, especially through deep potential molecular dynamics (DPMD), offer a promising alternative that closely aligns with the accuracy of first-principles methods. Our research used DPMD to study how salts affect water by comparing its results with ab initio molecular dynamics, SPC/Fw, AMOEBA, and MB-Pol models. We studied water’s behavior in salt solutions by examining its spatiotemporally correlated movement. Our findings showed that each model’s accuracy in depicting water’s behavior in salt solutions is strongly connected to spatiotemporal correlation. This study demonstrates both DPMD’s advanced abilities in studying water-salt interactions and contributes to our understanding of the basic mechanisms that control these interactions.

## Teaser

Machine learning force fields reveal correlated water motion as the key to understanding salt-induced water diffusion anomalies.

## INTRODUCTION

Water, beyond being one of the most familiar substances to humankind, is also a central figure in the long history of physical chemistry, having anomalous properties that set it apart from simpler liquids (*1*). These properties arise from the tetrahedral arrangement and network interactions among water molecules. A prominent example of these anomalies is the long-standing debate regarding the existence of a liquid-liquid critical point (LLCP) in water (*2*). Experimentally investigating this deeply supercooled state of water is challenging due to the need to avoid rapid crystallization. This challenge has made computational methods using in silico water models increasingly popular. However, the reliability of these models is paramount, as they must accurately capture the interactions of water molecules relevant to the phenomena under study. For instance, the LLCP is not observed in certain models, although these models do not seriously error in predicting basic thermodynamic properties (*3*, *4*). This discrepancy between qualitative failures and quantitative agreements highlights the importance of choosing reliable and adequate water models for investigating such complex phenomena.

In addition to the LLCP, our understanding of water, particularly as a solvent, remains incomplete. A specific area of interest is elucidating the bifurcation in water diffusivity influenced by the type of salt present (i.e., kosmotropic and chaotropic salts) and its concentration. These terms denote two distinct effects on the ordering of water molecules (*5*). Kosmotropic ions are known to enhance the ordering of water molecules, whereas chaotropic ions disrupt this order. The origin of this tetrahedral order is typically linked to the strong hydrogen bonding among water molecules and their four nearest neighbors. However, experimental studies using femtosecond pump-probe spectroscopy have reported a negligible effect of ions on the hydrogen bonding network (*6*). If the influence on water’s behavior is not primarily through alterations in the hydrogen bonding network, then what drives these observed effects? Here, we propose to address this complexity through a spatiotemporal characterization, offering physical insights into the underlying mechanisms.

Although many contemporary water models capably represent the thermodynamic and dynamical properties of water within reasonable limits (*7*–*10*), crucial discrepancies emerge with the introduction of other chemical species. A notable instance is the study by Kim *et al.* (*11*), which demonstrated that most water models inadequately capture the dynamic behavior of water following salt addition. Subsequently, Ding *et al.* (*12*) used ab initio molecular dynamics (AIMD) to show that water’s dynamics could exhibit two distinct qualitative behaviors, hinting at the role of electronic diversity in modifying water dynamics. While the fluctuating charge–discrete charge transfer (FQ-DCT) model, accounting for charge transfer, successfully replicated the diffusivity variations induced by different ion types, it did not verify the existence of dynamic heterogeneity (*13*, *14*). Andreev *et al.*’s later work (*15*) with a coarse-grained ion model accurately reproduced water dynamics without evidencing dynamic heterogeneity, as assessed by the non-Gaussian parameter and the Stokes-Einstein relation. They demonstrated that simple reparameterization of the Lennard-Jones parameters, informed by Born solvation theory, yields correct thermodynamic and dynamical properties (*15*, *16*). Aligning force fields with specific thermodynamic properties, such as solvation free energy, has been a successful approach in many studies for refining molecular models, indicating its effectiveness as a criterion for model enhancement. For instance, in the realm of all-atom force field reparameterizations, using osmotic pressure measurements stands out as a notable example. This particular adjustment has made contributions to biochemical simulations, illustrating the importance of thermodynamic data in improving the predictive accuracy of computational models (*17*, *18*). While classical force fields offer important insights, their simplifications and the omission of dynamic charge effects can skew our understanding of water’s true behavior. Successfully reparameterizing a model to accurately match a specific property is an important achievement. However, such corrections, until rigorously tested across a wide range of properties, remain unverified in their ability to comprehensively represent water’s complex behavior. This situation highlights the critical need for more sophisticated models, which not only seek to bridge these gaps but also respect and build upon the foundational efforts in force field development. Ensuring a model’s accuracy across the full spectrum of water’s properties is essential for advancing our understanding and accurately reflecting its multifaceted nature.

Machine learning force fields (MLFFs) have made noteworthy progress into diverse scientific areas such as material science, soft condensed matter, and physical chemistry, demonstrating benefits in various applications (*19*–*23*). In specific scenarios, MLFF models achieve prediction accuracy comparable to density functional theory (DFT) and other first-principles methods that supplied their training data (*24*–*26*). Although molecular dynamics (MD) simulations based on classical force fields may fall short in accurately replicating dynamical properties, they offer a notable speed advantage over first-principles methods, being more than six orders of magnitude quicker for systems comprising a few hundred atoms. In addition, the computational costs of MD simulations scale linearly with atom count, in contrast to the cubic scaling inherent to DFT, one of the foremost first-principles methods (*20*, *27*). Leveraging these strengths, MLFF is now being applied to explore complex physicochemical properties of water, such as the LLCP. It achieves precision and depth akin to first-principles calculations and extends these capabilities to larger scales without compromising the detailed chemical descriptions typically associated with ab initio methods (*28*–*34*). In this study, we use deep potential MD (DPMD), a popular MLFF model, to investigate water dynamics within salt solutions. Our approach includes a comparative analysis of DPMD against other established methods for simulating water and ion dynamics, such as MD with a flexible variant of the simple point charge model (SPC/Fw) (*35*) and Joung-Cheatham model (JC) (*36*), atomic multipole optimized energetics for biomolecular applications model (AMOEBA) (*37*, *38*), ab initio molecular dynamics (AIMD) {Becke-Lee-Yang-Parr functional with dispersion correction [BLYP (*39*, *40*)–D3 (*41*)]}, and many-body polarizable model (MB-Pol) (*42*–*44*).

## RESULTS AND DISCUSSION

**Fig. 1.**
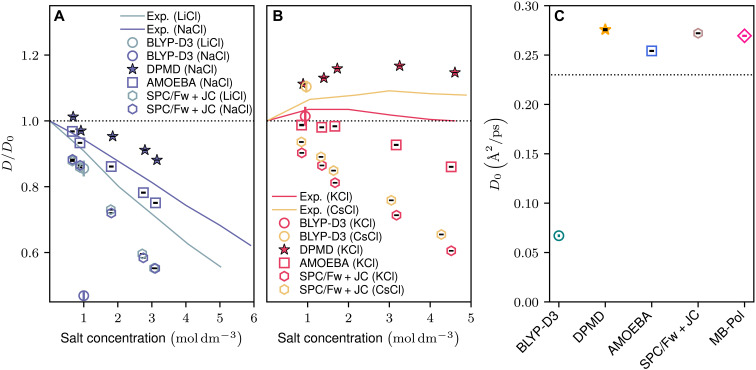
Comparison of water diffusion properties in salt solutions across various simulation models and experimental benchmarks. (**A**) Diffusivity of water in LiCl and NaCl solutions, featuring kosmotropic cations Li^+^ and Na^+^, across a range of concentrations. Each model’s diffusivity [AIMD (BLYP-D3), DPMD, AMOEBA, and SPC/Fw + JC] is normalized to each model’s respective pure water diffusivity, alongside experimentally measured values (*5*). (**B**) Similarly, water diffusivity in KCl and CsCl solutions, containing chaotropic cations K^+^ and Cs^+^, is presented to show concentration-dependent behavior, with normalization as described for (A). (**C**) Comparison of the pure water diffusivity across models, with a dotted line indicating experimental water diffusivity at 298.15 K (*79*). Note that bars on the markers represent standard errors, which are smaller than the marker size except for AIMD (BLYP-D3) and are thus not displayed for diffusivity henceforth.

The selection of models encompasses a broad range of the most commonly used water and ion simulations, providing a qualitative overview. As depicted in Fig. 1A, salt solutions with kosmotropic cations, Li^+^ and Na^+^, exhibit a deceleration in water dynamics as the concentration increases, a phenomenon observed across all considered models. This trend is in agreement with the findings reported by Kim *et al.* (*11*), although the extent of quantitative agreement differs among models. Particularly, the AIMD model using the BLYP-D3 functional (hereinafter BLYP-D3) shows notable deviations. From Fig. 1C, it is observed that simulations of pure water diverge from experimental benchmarks, a common challenge with many DFT functionals attributed to overstructuring, which could lead to an overestimation of salt’s impact on water dynamics. This discrepancy is partially linked to the absence of nuclear quantum effects (NQEs) in classical MD simulations. Recognizing the nuanced debate surrounding NQE’s role in momentum underestimation, we cautiously adopt the approach of some studies that suggest adjusting the simulation temperature by 10% (*45*, *46*) as a pragmatic solution. Accordingly, we set our thermostat to 330 K for simulations using the DPMD model while maintaining ambient temperature for other systems. However, as discussed in the Supplementary Materials, the main argument is not constrained by temperature.

Contrastingly, although models such as SPC/Fw + JC and AMOEBA do not qualitatively capture the dynamics, BLYP-D3 and DPMD accurately reflect the observed trends in solutions with chaotropic cations, K^+^ and Cs^+^, demonstrated in Fig. 1B. This replication of behavior, aligning with prior reports (*12*, *32*, *33*), supports our findings. As Avula *et al.* (*32*) have highlighted, altering the energetic landscape around ions, especially by changing the energy barrier for the freeing or escape of water from the solvation shell, provides a viable explanation for observed diffusion acceleration. Yet, describing the precise relationship between changes in water’s exchange behavior between the solvation shell and the bulk and its consequent effect on overall water dynamics remains an intricate challenge. More fundamentally, the investigation into how water dynamics shifts when the assumption of dynamic heterogeneity no longer applies is an intriguing question that our study seeks to explore further.

**Fig. 2.**
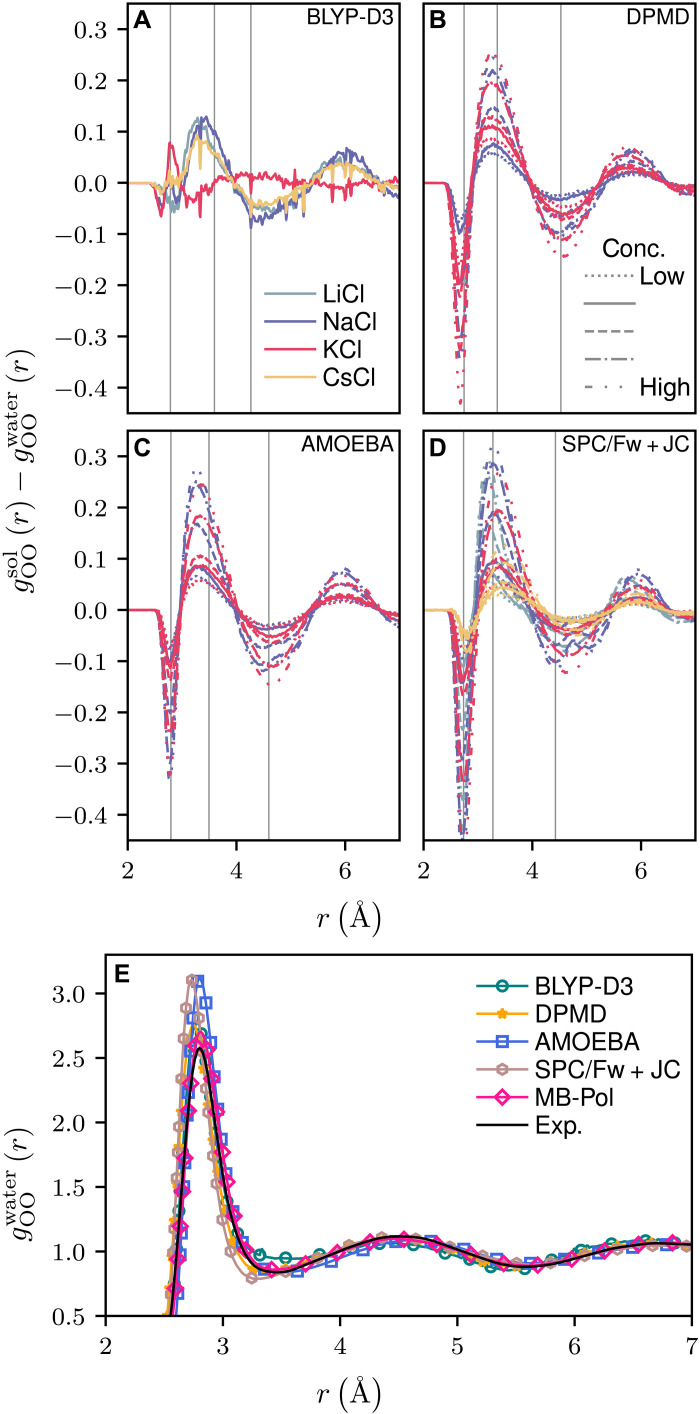
Differential oxygen-oxygen RDF $\left[g_{\text{OO}}^{\text{sol}},\left(r\right),-,g_{\text{OO}}^{\text{water}},\left(r\right)\right]$ between salt solution and pure water for different models. (**A**) AIMD (BLYP-D3), (**B**) DPMD, (**C**) AMOEBA, and (**D**) SPC/Fw + JC, along with (**E**) the oxygen-oxygen radial distribution function (RDF) of pure water for these models as well as MB-Pol and experimental data (*80*). Three vertical gray lines in each panel indicate, from left to right, the first peak, the first minimum, and the second peak of the RDF, derived from the pure water state of each model. The sequence of concentrations depicted adheres to the arrangement presented in Fig. 1. Note that the color assigned to each salt species is maintained consistently throughout all figures

In our endeavor to identify distinct “signatures” that could differentiate between models based on their ability to reproduce experimental observations, we commence our investigation by examining the static structure of water. Specifically, we focus on the radial distribution function (RDF) between oxygen atoms, denoted as $g_{\text{OO}}\left(r\right)$. Figure 2 provides a comparative analysis of how various salts at different concentrations influence the structure of water, illustrated by the differential RDF [$g_{\text{OO}}^{\text{sol}}\left(r\right)-g_{\text{OO}}^{\text{water}}\left(r\right)$].

Analysis across Fig. 2 (A to D) reveals an absence of a consistent trend in water’s static structure along the Hofmeister series (Li^+^ < Na^+^ < K^+^ < Cs^+^). This observation aligns with existing literature that suggests a negligible impact of ions on the hydrogen bonding network of water (*6*). Common to all models is a diminished structuring around the first solvation shell, evidenced by a reduction in the first peak height of the RDF. Moreover, the structuring beyond this immediate neighborhood also decreases, as indicated by a less pronounced second peak and a more leveled profile following the first peak. Full RDF profiles can be found in the Supplementary Materials.

For instance, the BLYP-D3 model (Fig. 2A) exhibits similar shifts in the RDF upon the addition of LiCl, NaCl, and CsCl, despite great differences in their diffusivities. Notably, KCl emerges as an outlier, which may be due to the inherent limitations of first-principles BLYP-D3 simulations, such as potentially insufficient sampling, relaxation time, and system size within the constraints of available computational resources. The DPMD model’s scenario (Fig. 2B) also shows negligible difference between the effects of NaCl and KCl. In addition, it remains ambiguous whether a direct correlation exists between concentration levels and RDF profile trends. For example, despite the diffusivity of KCl plateauing or even decreasing at higher concentrations, the RDF deformation continues to intensify with concentration.

For the AMOEBA (Fig. 2C) and SPC/Fw + JC (Fig. 2D) models, there is a notable correlation between the degree of reduction in water structuring (under-structuring) and the extent of decreased slowdown in water dynamics. However, these models invariably exhibit slower water dynamics with the addition of salt. Hence, although the reduction in water structuring may hint at the extent to which water dynamics are slowed, it does not clarify how the specific character of ions, whether chaotropic or kosmotropic, influences water dynamics.

Exploring water reorientation dynamics (*47*–*51*) or the entropic impacts of salts (*52*, *53*) is an interesting direction for further research. Our focus, however, is drawn toward the cooperative motion of water molecules. Recent studies using mode coupling theory (MCT) on salt solutions, such as the work by Kumar and Bagchi (*54*), suggest that an emphasis on the time-dependent correlated dynamics of water could provide a valuable framework. This perspective is consistent with the observed cooperativity in water dynamics (*51*, *55*) and glasslike behavior (*56*). However, it does not necessarily imply the presence of the same level of strongly correlated dynamics and heterogeneity found in supercooled water (*57*–*59*).

To further explore this cooperative aspect of water dynamics, we calculated the four-point susceptibility of water’s translational motion ($\chi_{4}$), thus bifurcating our analysis into relaxation time ($t_{\text{relax}}$) and correlation length ($\xi_{4}$).

For MD simulations involving $N_{O}$ water molecules, the relaxation order parameter, Q(t), is estimated as follows (*60*)

$$\begin{array}{cc} Q\left(t\right) & =\sum_{i=1}^{N_{O}}\sum_{j=1}^{N_{O}}w\left(\left|r_{\mathrm{ij}}-\mu_{j}\right|\right)\\ & =Q_{S}\left(t\right)+Q_{D}\left(t\right)\\ & =\sum_{i=1}^{N_{O}}w\left(\mu_{i}\right)+\sum_{i=1}^{N_{O}}\sum_{j\neq i}^{N_{O}}w\left(\left|r_{\mathrm{ij}}-\mu_{j}\right|\right) \end{array}$$ (1)

where the overlap function, w, is defined as w(r)≡Θ(a−r), $r_{\mathrm{ij}}\equiv r_{j}(0)-r_{i}\left(0\right)$, $\mu_{i}\equiv r_{i}\left(t\right)-r_{i}\left(0\right)$, and a is a length scale with Θ being the Heaviside step function.

**Fig. 3.**
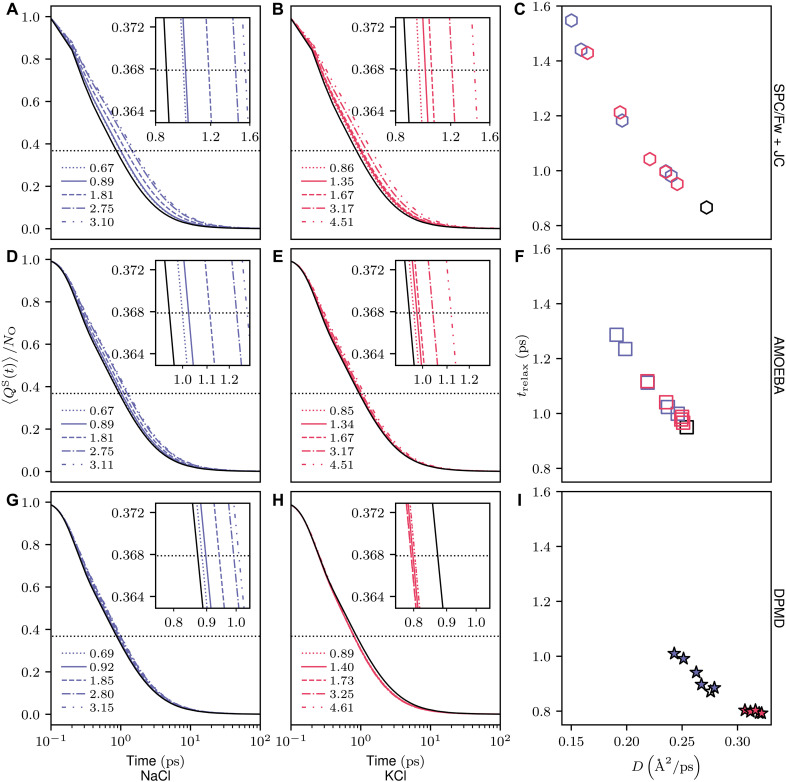
Relaxation behavior of water in NaCl and KCl solutions at various concentrations, analyzed through different models. (**A** to **C**) SPC/Fw + JC, (**D** to **F**) AMOEBA, and (**G** to **I**) DPMD illustrate the normalized relaxation order parameter $\langle Q_{S}\left(t\right)\rangle /N_{O}$, revealing the dynamic properties of water. [(C), (F), and (I)] The correlation between the estimated relaxation time, $t_{\text{relax}}$, and the diffusion coefficient. The black-edged markers without face color represent the pure water system throughout all figures. Note that the legend in each panel denotes the concentration.

Q(t) measures the overlapping of two configurations separated by time t. The Q(t) can be decomposed into two terms, $Q_{S}$ and $Q_{D}$. Starting from a unity, the former, self part, measures the molecules diffusing less than a length, a=1 Å, which is contribution from the localization of particles. When it goes to zero, all particles diffuse over the length. On the other hand, the latter, the distinct part measures the contribution from the replacement of neighboring molecules. When the replacement of neighbor particles occurs, it goes to zero. The choice of only picking up $Q_{S}$ out of $Q\left(t\right)=Q_{S}\left(t\right)+Q_{D}\left(t\right)$ can be justified by the report $Q_{D}$ does not contribute much to later-discussed four-point susceptibility $\chi_{4}\left(t\right)$ (*60*–*63*). Considering these points, this study focuses on $Q_{S}$, aligning with methodologies applied in previous four-point correlation analyses for supercooled water (*59*). We investigate the alteration in relaxation dynamics upon the addition of salt by quantifying the relaxation time $t_{\text{relax}}$ through the equation $\langle Q_{S}\left(t=t_{\text{relax}}\right)\rangle /N_{O}=e^{-1}$. As illustrated in Fig. 3 (A to C), the SPC/Fw + JC model consistently exhibits prolonged relaxation times ($t_{\text{relax}}$) for both NaCl and KCl salts. This observation is intuitively aligned with the premise that an acceleration in the relaxation correlates with a reduction in translational diffusion. For the AMOEBA model (Fig. 3, D to F), we can see the same extended relaxation time. This observation aligns with the intuitive premise that a deceleration in relaxation corresponds to a reduction in translational diffusion. While these models can only capture the slowdown in water dynamics upon salt addition, they do not refute the hypothesis that enhanced dynamics may stem from expedited relaxation processes.

In contrast, the DPMD model reveals a distinct behavior. NaCl solutions consistently exhibit longer $t_{\text{relax}}$ compared to pure DPMD water, whereas KCl solutions display accelerated relaxation times. This unique ability of the DPMD model to accurately reproduce the acceleration of water dynamics with the addition of chaotropic salts highlights a key qualitative difference in capturing anomalous water dynamics across various models. The DPMD model also reveals two nuanced observations: (i) At the lowest concentration, a NaCl sample unexpectedly exhibits faster dynamics than pure water, and (ii) the relaxation time remains nearly unchanged across varying concentrations of KCl. While these minor discrepancies might appear as statistical anomalies, further discussion in subsequent sections will reveal their connection to the underlying dynamics.

It is also noteworthy to mention that we do not perform estimations for BLYP-D3 and MB-Pol, which are shown in Fig. 2E. The exclusion of BLYP-D3 is due to difficulties in achieving reliable statistical analyses, unlike with static structure or diffusivity measurements, where the statistical errors are minor in comparison to the marker sizes used. This issue is particularly relevant for $\chi_{4}$ estimation in BLYP-D3 systems, typically consisting of around a hundred water molecules, where the available timescale for analysis is notably limited. This situation has allowed us to highlight a key advantage of MLFF as an alternative to first-principles calculations. It not only establishes itself as a viable approach but also advances our understanding of water dynamics. This is achieved by efficiently managing the computational demands of large-scale simulations while retaining the valuable insights of first-principles theories.

Furthermore, MB-Pol stands out for its high physicochemical accuracy in water modeling (*64*), showing compatibility with both alkali metals and halide ions as indicated in MB-nrg (*65*, *66*). However, the current lack of a model that can incorporate both ion types simultaneously presents a considerable challenge. Although exploring the impact of introducing a single ion type without its counter ion, as investigated in the FQ-DCT study, is promising, it is outside the current scope of our research but may provide a valuable direction for future studies.

We turn our attention to the four-point susceptibility ($\chi_{4}$), which, as derived from Eq. 1, can be expressed as follows

$$\begin{array}{cc} \chi_{4}\left(t\right) & =\frac{\beta V}{N^{2}}\left[\langle Q^{2}\left(t\right)\rangle -{\langle Q\left(t\right)\rangle }^{2}\right]\\ & =\frac{\beta V}{N^{2}}\left[\langle {\left[Q_{S}\left(t\right)+Q_{D}\left(t\right)\right]}^{2}\rangle -{\langle \left[Q_{S}\left(t\right)+Q_{D}\left(t\right]\right)\rangle }^{2}\right]\\ & =\frac{\beta V}{N^{2}}\left[\langle Q_{S}^{2}\left(t\right)\rangle -{\langle Q_{S}\left(t\right)\rangle }^{2}\right]\\ & +\frac{2\beta V}{N^{2}}\left[\langle Q_{S}\left(t\right)Q_{D}\left(t\right)\rangle -\langle Q_{S}\left(t\right)\rangle \langle Q_{D}\left(t\right)\rangle \right]\\ & +\frac{\beta V}{N^{2}}\left[\langle Q_{D}^{2}\left(t\right)\rangle -{\langle Q_{D}\left(t\right)\rangle }^{2}\right]\\ & =\chi_{4}^{\text{SS}}\left(t\right)+2\chi_{4}^{\text{SD}}\left(t\right)+\chi_{4}^{\text{DD}}\left(t\right) \end{array}$$ (2)

In our approach, we focus on isolating the self-part component $\chi_{4}^{\text{SS}}\left(t\right)$ from the overall four-point susceptibility, $\chi_{4}\left(t\right)$, as outlined in Eq. 2. This can be justified by the fact that, as we discussed above, $Q_{S}\left(t\right)$ is a major contribution to $\chi_{4}$. Thus, taking only $Q_{S}\left(t\right)$ out of Q(t) neglects contributions from the interference ($\chi_{4}^{\text{SD}}$) and discrete ($\chi_{4}^{\text{DD}}$) components. The key parameters derived from $\chi_{4}^{\text{SS}}\left(t\right)$ are the peak time $t_{\text{peak}}$, where the four-point susceptibility reaches its maximum, and the peak height $\chi_{4}^{\text{SS}}\left(t=t_{\text{peak}}\right)$. It is important to note that both $t_{\text{peak}}$ and $t_{\text{relax}}$ are related to the same temporal scale, demonstrating a direct correspondence between them, as elaborated in the Supplementary Materials. The peak height of the four-point susceptibility is generally considered indicative of the spatial dynamics correlation length within glass, as corroborated by several studies (*67*–*70*).

**Fig. 4.**
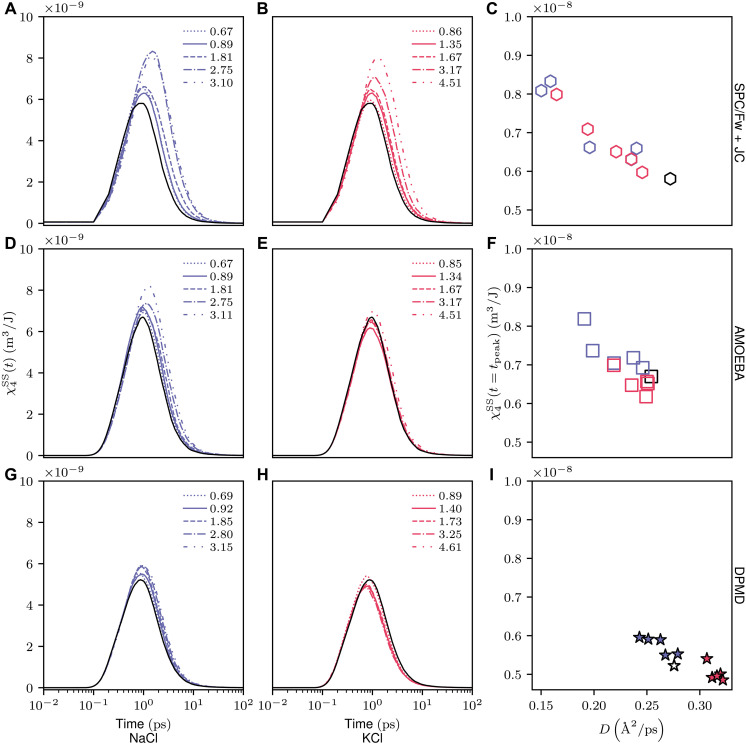
Four-point susceptibility of water at varied concentrations in NaCl and KCl solutions, analyzed through three computational models. (**A** to **C**) SPC/Fw + JC, (**D** to **F**) AMOEBA, and (**G** to **I**) DPMD. [(C), (F), and (I)] The correlation between diffusivity and the peak height of the four-point correlation function, indicative of the correlation length ($\xi_{4}$). Note that the legend in each panel denotes the concentration.

The addition of NaCl in the SPC/Fw + JC (Fig. 4, A to C) and AMOEBA (Fig. 4, D to F) models leads to extended correlation time and length, corresponding to the upper and right direction shift of the peaks in the four-point susceptibility, respectively. However, the AMOEBA model displays a more complicated behavior for KCl. At the highest concentration, the AMOEBA KCl solution exhibits a higher peak height than pure water, but, at other concentrations, its height is lower than that of pure water. This observation might be related to the experimentally observed suppression of enhanced water diffusivity in high-concentration KCl solutions, as shown in Fig. 1A. Similar trends can be observed in the DPMD model, where NaCl results in a higher peak compared to pure water, while KCl leads to a lower peak. The DPMD model also shows an incoherent enhanced peak height in the KCl solution at the lowest concentration. These findings suggest that there is a spectrum in the capabilities of different models to describe the spatiotemporal correlation of water dynamics, which depends on the salt type and concentration.

In addition to the non-monotonic behavior observed in KCl solutions, the DPMD model reveals a slightly faster water dynamics at the lowest concentration in NaCl solution (Fig. 4I), despite the enhanced $\chi_{4}^{\text{SS}}\left(t=t_{\text{peak}}\right)$ at all concentrations. Considering the outliers observed in both relaxation time and correlation length, it appears that neither of these analyses fully explains diffusivity. We propose that a better understanding of diffusivity can be obtained through an integrated perspective of both length scale and timescale.

**Fig. 5.**
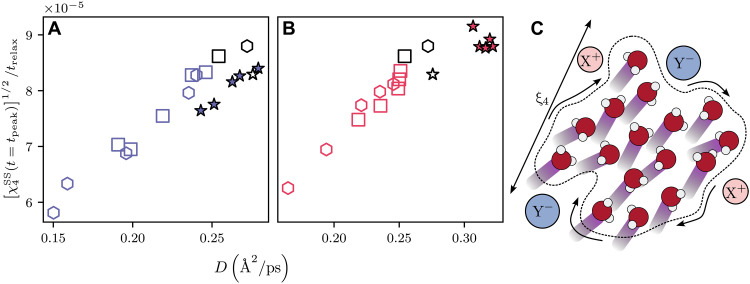
Correlation-scaled diffusivity, ${\left[\chi_{4}^{\text{SS}}\left(t=t_{\text{peak}}\right)\right]}^{1/2}/t_{\text{relax}}$, as a function of D. Refer to Fig. 3 for symbol definitions. (**A**) Results for NaCl solutions. (**B**) Results for KCl solutions. (**C**) A schematic representation of the correlated water dynamics observed in the system.

If the diffusion in salt solutions is governed by the collective behavior of water molecules, as suggested by several studies (*51*, *54*, *55*), then the diffusivity (D) should be intimately related to the characteristic length scale and timescale associated with these collective dynamics. An intuitive picture of this collective behavior involves groups of water molecules moving in a correlated manner, as illustrated schematically in Fig. 5C. A further schematic representation of how clusters of correlated water molecules emerge over time, related to the four-point susceptibility, can be found in the Supplementary Materials. We propose that D can be expressed as a function of the correlation length ($\xi_{4}$) and the relaxation time ($t_{\text{relax}}$), following the scaling relation $D=D\left(\xi_{4},t_{\text{relax}}\right)∼\xi_{4}^{2}/t_{\text{relax}}$.

$\chi_{4}\left(t\right)$ is known to capture the number particles with correlated dynamics within a time frame t and its peak height related to the number of correlated molecules (*71*, *72*). The correlation length $\xi_{4}$ is related to the peak height of the four-point susceptibility $\chi_{4}^{\text{SS}}\left(t=t_{\text{peak}}\right)$ through a scaling exponent ζ, such that $\chi_{4}^{\text{SS}}\left(t=t_{\text{peak}}\right)∼\xi_{4}^{\zeta }$. In the context of glass-forming liquids, ζ typically ranges from 2 to 4, depending on the geometric nature of the correlations (*67*). If $\chi_{4}\left(t=t_{\text{peak}}\right)$ is directly a measure of the number of correlated molecules and if a compact region, i.e., a region without much fractality, is assumed, then the volume of the correlated region scales as $V∼\xi^{3}$, leading to ζ = 3 (*67*, *70*). We find that empirically adopting ζ = 4 yields a clearer proportionality between $\chi_{4}^{\text{SS}}\;{\left(t=t_{\text{peak}}\right)}^{\;2/\zeta }/t_{\text{relax}}$ and D. Consequently, we use = 4, leading to the formula $D∼{\left[\chi_{4}^{\text{SS}}\left(t=t_{\text{peak}}\right)\right]}^{1/2}/t_{\text{relax}}$, which we define as the correlation-scaled diffusivity, as illustrated in Fig. 5 (A and B).

This scaling concept provides a unified framework to interpret the intriguing observations related to $t_{\text{relax}}$ and $\chi_{4}^{\text{SS}}\left(t=t_{\text{peak}}\right)$ in salt solutions of KCl and NaCl. Notably, a higher diffusivity can arise either from a longer $t_{\text{relax}}$ or a lower $\chi_{4}^{\text{SS}}\left(t=t_{\text{peak}}\right)$, suggesting that the link between diffusivity and these quantities is nuanced and involves a delicate balance. For instance, in low-concentration NaCl solutions, the larger $\chi_{4}^{\text{SS}}\left(t=t_{\text{peak}}\right)$ compared to pure water is counterbalanced by a much longer $t_{\text{relax}}$, resulting in reduced (correlation-scaled) diffusivity. Conversely, low-concentration KCl solutions exhibit a higher $\chi_{4}^{\text{SS}}\left(t=t_{\text{peak}}\right)$ than pure water but with a shorter $t_{\text{relax}}$, leading to decreased diffusivity and correlation-scaled diffusivity. Thus, the correspondence between diffusivity and correlation-scaled diffusivity effectively captures the intricate interplay between $t_{\text{relax}}$ and $\chi_{4}^{\text{SS}}\left(t=t_{\text{peak}}\right)$ in governing the correlated dynamics of these systems.

In addressing the challenge identified by Kim *et al.* (*11*), regarding the persistent difficulty in simulating water diffusivity in salt solutions via in silico models, our study offers physical insights into the mechanisms through which salts alter water dynamics. Specifically, we introduce a framework that incorporates both correlation length ($\xi_{4}$) and relaxation time ($t_{\text{relax}}$) into an alternative metric: correlation-scaled diffusivity. This metric enables a nuanced interpretation of diffusivity, revealing it as a product of two distinct factors: correlation length and time. Our findings highlight that, in low-concentration salt solutions, water dynamics exhibit nonlinear behaviors in both $\xi_{4}$ and $t_{\text{relax}}$, underscoring the importance of considering these parameters together through correlation-scaled diffusivity to fully grasp the complexity of water’s behavior in salt solutions.

Moreover, our work benefits greatly from the advancements in the field of MLFF, particularly the DPMD model (*20*). The application of DPMD, one of the most popular MLFF for water, has proven instrumental in accurately reproducing water diffusivity (*32*, *33*). Our analysis extends these successes by providing a fresh perspective on water dynamics in salt solutions, contrasting our findings with those derived from qualitatively different models and thus offering a clear delineation of the source of disparate behaviors in water dynamics. This comparison not only demonstrates the superiority of DPMD but also opens the door to deeper explorations into the spatiotemporal correlations of water molecules in diverse environments.

Looking ahead, this work opens up several promising avenues for future research endeavors. While our analysis did not extensively scrutinize the determination of the scaling exponent ζ, which is related to the geometric nature of the correlations, a detailed investigation of the dynamic geometric structure of water molecules could yield valuable insights. Such an exploration could unravel the intricate relationship between the structural and dynamic aspects of water’s collective behavior.

In addition, probing the temperature-dependent transitions between the kosmotropic and chaotropic behavior of salts (*11*, *73*, *74*) could further elucidate the nuances of salt-water interactions and their impact on the correlated dynamics. As the system approaches deeply supercooled states, an intricate interplay between the correlation length and relaxation time may emerge, potentially unveiling different dynamics regimes governed by the collective behavior of water molecules.

Another intriguing direction for future research lies in the realm of water’s reorientation dynamics (*47*–*51*). By extending our analysis to incorporate orientational four-point susceptibility (*59*), it may be possible to gain deeper insights into the collective reorientation of water molecules, a phenomenon that has been recently highlighted in a study (*51*). Such an investigation could shed light on the interplay between translational and rotational dynamics in these systems, potentially revealing undiscovered facets of the collective behavior.

In summary, this work demonstrates the power of MLFFs in uncovering the intricate dynamics of water molecules in salt solutions. By leveraging the capabilities of MLFFs, we have gained physical insights into the complex interplay between water and dissolved ions, shedding light on the fundamental aspects that govern the dynamical behavior of these systems. Our findings not only contribute to the advancement of knowledge in the field of aqueous salt solutions but also underscore the immense potential of data-driven approaches in scientific discovery.

## MATERIALS AND METHODS

### Molecular dynamics

MD simulations were performed using various water and salt models, including SPC/Fw (*35*) + JC (*36*), AMOEBA (*37*, *38*), AIMD [BLYP (*39*, *40*)–D3 (*41*)], and MB-Pol (*42*–*44*). Initial configurations were prepared using PACKMOL (*75*) at a lower density than that of water. For computationally intensive models, classical MD simulations were used in the initial stage of the protocol to facilitate faster equilibration. The systems were equilibrated under isobaric-isothermal (NPT) conditions, followed by canonical ensemble (NVT) simulations. Production trajectories were then collected for analysis in the NVT ensemble.

Except for AIMD, all systems were simulated using either LAMMPS (*76*) or OpenMM (*77*), depending on the specific requirements of the force field and its suitability for processing in the workflow. For AIMD, we used CP2K (*78*). Some models were implemented or intended to run on graphics processing unit (GPU), while others were not implemented to run on GPU and could only run on central processing unit (CPU). The speed across CPU and GPU equipment was tested, and the faster approach was chosen for each model. The specific simulation protocols and system configurations are detailed in the Supplementary Materials.

### Structural and dynamical characterization

The static geometric structure of water (oxygen) was characterized by calculating the oxygen-oxygen RDF. To investigate the spatiotemporal volume of correlated dynamics of water molecules, the self part of four-point susceptibility ($\chi_{4}^{\mathrm{SS}}$), derived from relaxation profiles [Q(t)] of water molecules, was estimated (Eq. 2). The temperature dependence of water dynamics was also investigated for pure water systems using DPMD, MB-Pol, and SPC/Fw + JC models to compare the capabilities of each model. Correlation times were quantified using the peak time of $\chi_{4}^{\mathrm{SS}}$ ($t_{\text{peak}}$) and the relaxation time of the relaxation function ($t_{\text{relax}}$), and their correspondence was also evaluated. Please refer to the Supplementary Materials for additional results on temperature dependency and the correspondence between the two timescales.

## Supplementary Materials

### The PDF file includes:

Supplementary Text Tables S1 to S5 Figs. S1 to S9 References
