# Supplementary material for: Spatiotemporal characterization of water diffusion anomalies in saline solutions using machine learning force field
Source: Sci Adv. 2024 Dec 11;10(50):eadp9662. doi: 10.1126/sciadv.adp9662 (PMC11633738; doi:10.1126/sciadv.adp9662)
Supplement: Supplementary file 1 — Supplementary Text Tables S1 to S5 Figs. S1 to S9 References [file sciadv.adp9662_sm.pdf]

Science Advances
AAAS

Supplementary Materials for

Spatiotemporal characterization of water diffusion anomalies in saline solutions using machine learning force field

Ji Woong Yu *et al.*

Corresponding author: Won Bo Lee, wblee@snu.ac.kr; Tae Jun Yoon, tyoon124@snu.ac.kr

Sci. Adv. **10**, eadp9662 (2024)
DOI: 10.1126/sciadv.adp9662

The PDF file includes:

Supplementary Text
Tables S1 to S5
Figs. S1 to S9
References

Other Supplementary Material for this manuscript includes the following:

Movie S1

Preparation

This section describes how we prepared the dynamic and structural data of different water/salt models.

BLYP-D3

The ab-initio molecular dynamics (AIMD) simulations were performed by utilizing the Becke, Lee, Yang, and Parr (BLYP) exchange-correlation functional (*39, 40*) coupled with the DFT-D3 dispersion (*41*) correction. The initial configuration was prepared by using classical molecular dynamics simulations in an isobaric-isothermal (NPT) environment, employing the SPC/E+JC force field via OpenMM (*77*). This initial step was followed by transferring the resulting snapshot to CP2K (*78*), where all subsequent simulations were conducted. The snapshot underwent energy minimization using the BLYP-D3 functional, with a Double Zeta Valence with Polarization (DZVP) basis set and Goedecker-Teter-Hutter (GTH) pseudopotentials for the DFT calculations. A brief NPT simulation, lasting at least 10ps with a timestep of 0.5fs, was then conducted. The average volume from the final 5ps of this simulation was used to set up a canonical ensemble (NVT) simulation. The NVT simulation phase yielded a 100ps trajectory. We maintained consistent conditions of $P = 1$bar and $T = 300$K for all NPT simulations, and a temperature of $T = 300$K for all NVT simulations.

DPMD

The DPMD model, as provided in the work by Zhang et al. (*81*), was employed in our simulations. The model was trained using the strongly constrained and appropriately normed (SCAN) functional DFT data. This model's thermodynamic validity is thoroughly discussed in their work. Initial configurations were generated using Packmol (*75*) and then equilibrated through NPT molecular dynamics (MD) simulation for 500ps. The cell size for subsequent NVT runs was determined using the average volume calculated from the last 1ps of the equilibration phase. Following a relaxation period of 100ps in an NVT environment, production trajectory data were collected from a 1ns NVT simulation. All simulations were conducted using LAMMPS (*76*). For salt solution simulations, conditions were set to 1bar and $T = 330$K for NPT environments and maintained at $T = 330$K for NVT runs. In the case of pure water simulations, two temperatures, $T = 300$K and $T = 330$K, were tested to analyze variations in χ_4.

The full details of training, validation, and benchmarking can be found in the supplementary information of Zhang et al. (*81*). Here we present a summarized iteration of those contents.

As the training of KCl and NaBr follows the same procedure as NaCl, we only explain the training of NaCl. The NaCl DPMD model is first initialized using $\sim$ 4000 NaCl configurations and $\sim$ 1000 pure water configurations from ab-initio molecular dynamics. The snapshots are generated using Quantum Espresso (*82*). The strongly constrained and appropriately normed (SCAN) functional (*83*) is chosen for exchange-correlation. Hamann-Schluter-Chiang-Vanderbilt (HSCV) pseudopotentials (*84, 85*) are used for Oxygen, Hydrogen, and Chloride, while the Norm-Conserving Vanderbilt (ONCV) pseudopotential (*86*) is used for the sodium.

The training process follows an iterative procedure where four independent deep neural network models are trained using the DeePMD-kit package (*87*). Molecular dynamics simulations are then conducted to explore new configurations, identifying those with high uncertainty, $\zeta > 0.15$ eV/Å where $\zeta = \max_i \sqrt{\langle \| \boldsymbol{F}_i - \bar{\boldsymbol{F}} | \rangle}$ and $\bar{\boldsymbol{F}}$ is the average force of four different models for the i-th atom. Note that ζ is different from the exponent in the main manuscript. Here, ζ denotes the maximum standard deviation of the predicted atomic forces across the model. These uncertain configurations undergo DFT calculations and are added to the training set. This process repeats until less than 0.005% of configurations have high uncertainty, ensuring a well-converged model.

Validation of the DPMD model involves several comprehensive steps. Comparison with DFT calculations on 180 configurations shows good agreement, with RMSEs of 2.57×10^{-4} eV/atom for energy and 7.24×10^{-2} eV/Å for forces. The model's radial distribution functions closely match AIMD results (*45*), while comparisons with Deep Potential with Long-Range correction (DPLR) (*88*) validate the 6 Å cutoff used for interactions. System size effects are assessed through simulations with up to 4096 water molecules, demonstrating converged structure factors.

The model's performance is further validated against experimental data. Density predictions for both pure water and NaCl solutions agree with experimental trends (*89*), showing less than 5% error. Calculated structure factors also show good agreement with neutron diffraction data (*90, 91*), particularly for NaCl and KCl solutions. This extensive validation demonstrates that the DPMD model accurately reproduces both DFT results and experimental measurements.

AMOEBA

AMOEBA (*37, 38*) is an advanced polarizable force field that employs atomic multipoles up to quadrupole moments to describe permanent electrostatic interactions, surpassing traditional fixed partial charge models. It implements polarizability through a sophisticated self-consistent induced dipole model, where each atom is assigned a polarizability value. Dipoles are induced in response to the electric field generated by all other permanent multipoles and induced dipoles, iteratively solved until convergence is achieved.

The AMOEBA force field was adopted as a benchmark for polarization effects (*37, 38*), utilizing the TINKER parameter file (`amoebabio18.prm`) provided in TINKER (*92*) official github. In all simulations, a van der Waals cutoff of 10Å and a 64-particle mesh Ewald (PME) grid for each dimension were implemented. The real-space PME cutoff was established at 7Å. Packmol was employed to generate the initial configuration. Subsequently, the system underwent relaxation for 500ps in an NVT MD setup, followed by an extended relaxation period of at least 1ns + 100ps in an NPT MD simulation. The cell size for subsequent NVT simulations was determined based on the average volume of the last 100ps of this phase. Prior to embarking on the 1ns NVT production run, the system was further relaxed under the NVT ensemble for 500ps. All simulations were executed within the LAMMPS framework (*76*), with the integration timestep set at 1fs. A constant pressure of 1bar was maintained for all NPT simulations, and a temperature of $T = 300$K was applied across all simulation stages.

SPC/Fw+JC

SPC/Fw (*35*) is an enhanced version of the simple point charge (SPC) three-site water model, incorporating intramolecular flexibility that allows for bond stretching and angle bending. In this study, we simulated SPC/Fw in conjunction with the widely-adopted Joung-Cheatham (JC) (*36*) monovalent ion models to accurately represent saline solutions.

Initial configurations for the SPC/Fw (*35*)+JC (*36*) model were generated using Packmol. A 2ns NPT simulation was conducted, with the latter half utilized to determine the equilibrium

density. Following this, a 2ns NVT simulation was executed using the scaled box at the established equilibrium density, with its latter half designated as the production phase for subsequent analyses. All simulations were carried out within the LAMMPS software (*76*) with integration timestep of 1fs. For salt solution scenarios, NPT simulations were conducted at a temperature of 300K and pressure of 1atm, while NVT runs were also set at 300K. In the case of pure water (SPC/Fw) systems, identical protocols were followed, with the exception of executing simulations across a temperature range from $T = 240\text{K}$ to $T = 300\text{K}$.

MB-Pol

MB-Pol (*42, 43, 44*) represents a state-of-the-art water model constructed on the foundation of the many-body expansion of water's interaction energy. It explicitly incorporates one-body, two-body, and three-body interactions, along with a Thole-type model and dispersion effects. The model's parameters are derived from high-accuracy ab initio calculations, specifically using coupled cluster theory with single, double, and perturbative triple excitations (CCSD(T)), widely regarded as the gold standard in quantum chemistry.

As explained in the manuscript, MB-Pol (*42, 43, 44*) does not fully support salt solution here we considered at the moment. However, the fidelity of MB-Pol to experimental is very high so it is still worth testing as reference model only for pure water systems. We used two-body cutoff as $R_{2\text{B}} = 9\text{Å}$ and three-body cutoff as $R_{3\text{B}} = 4.5\text{Å}$ with dipole tolerance of 10^{-8}. The initial configurations were the last snapshots SPC/Fw pure water systems at each temperature. We ran 50ps of NPT run half of which was used to estimate the average density of relaxed water. After 200ps of NVT relaxation, we collected data from 1ns NVT simulation trajectory. For NPT runs, pressure was maintained at $P = 1\text{atm}$. For both NPT and NVT runs, temperature was thermostatted at different target temperatures ranging from $T = 240\text{K}$ to $T = 300\text{K}$.

System Configuration

The configuration of each salt solution is given in Tables S1 to S4 and configuration of pure water is given in Table S5. N_{ion} denotes the number of ion pairs, N_{water} denotes the number of water molecules, and concentration is given in mol L^{-1}

Table S1. System configuration for BLYP-D3

AIMD (BLYP-D3)											
LiCl			NaCl			KCl			CsCl		
N_{ion}	N_{water}	conc.	N_{ion}	N_{water}	conc.	N_{ion}	N_{water}	conc.	N_{ion}	N_{water}	conc.
2	120	1.00	2	120	1.00	2	120	0.94	2	120	0.97

Table S2. System configuration for DPMD

DPMD					
NaCl			KCl		
N_{ion}	N_{water}	conc.	N_{ion}	N_{water}	conc.
64	5312	0.69	64	4096	0.89
64	3968	0.92	128	5120	1.40
128	3840	1.85	128	4096	1.73
192	3712	2.80	256	4096	3.25
256	4352	3.15	384	4096	4.61

Table S3. System configuration for AMOEBA

AMOEBA					
NaCl			KCl		
N_{ion}	N_{water}	conc.	N_{ion}	N_{water}	conc.
64	5312	0.64	64	4096	0.82
64	3968	0.86	128	5120	1.29
128	3840	1.73	128	4096	1.60
192	3712	2.63	256	4096	3.01
256	4352	2.96	384	4096	4.25

Table S4. System configuration for SPC/Fw+JC

SPC/Fw+JC											
LiCl			NaCl			KCl			CsCl		
N_{ion}	N_{water}	conc.	N_{ion}	N_{water}	conc.	N_{ion}	N_{water}	conc.	N_{ion}	N_{water}	conc.
64	5312	0.67	64	5312	0.67	64	4096	0.86	64	4096	0.85
64	3968	0.89	64	3968	0.89	128	5120	1.35	128	5120	1.33
128	3840	1.79	128	3840	1.81	128	4096	1.67	128	4096	1.63
192	3712	2.72	192	3712	2.75	256	4096	3.17	256	4096	3.05
256	4352	3.06	256	4352	3.10	384	4096	4.51	384	4096	4.27

Table S5. System configuration for pure water systems

T (K)	Pure water				
	AIMD	DPMD	SPC/Fw	AMOEBA	MB-Pol
	N_{water}	N_{water}	N_{water}	N_{water}	N_{water}
330		2048			
300	120	2048	2048	4096	2048
280		2048	2048		2048
260		2048	2048		2048
240		2048	2048		2048

Oxygen-Oxygen Radial Distribution Function (RDF)

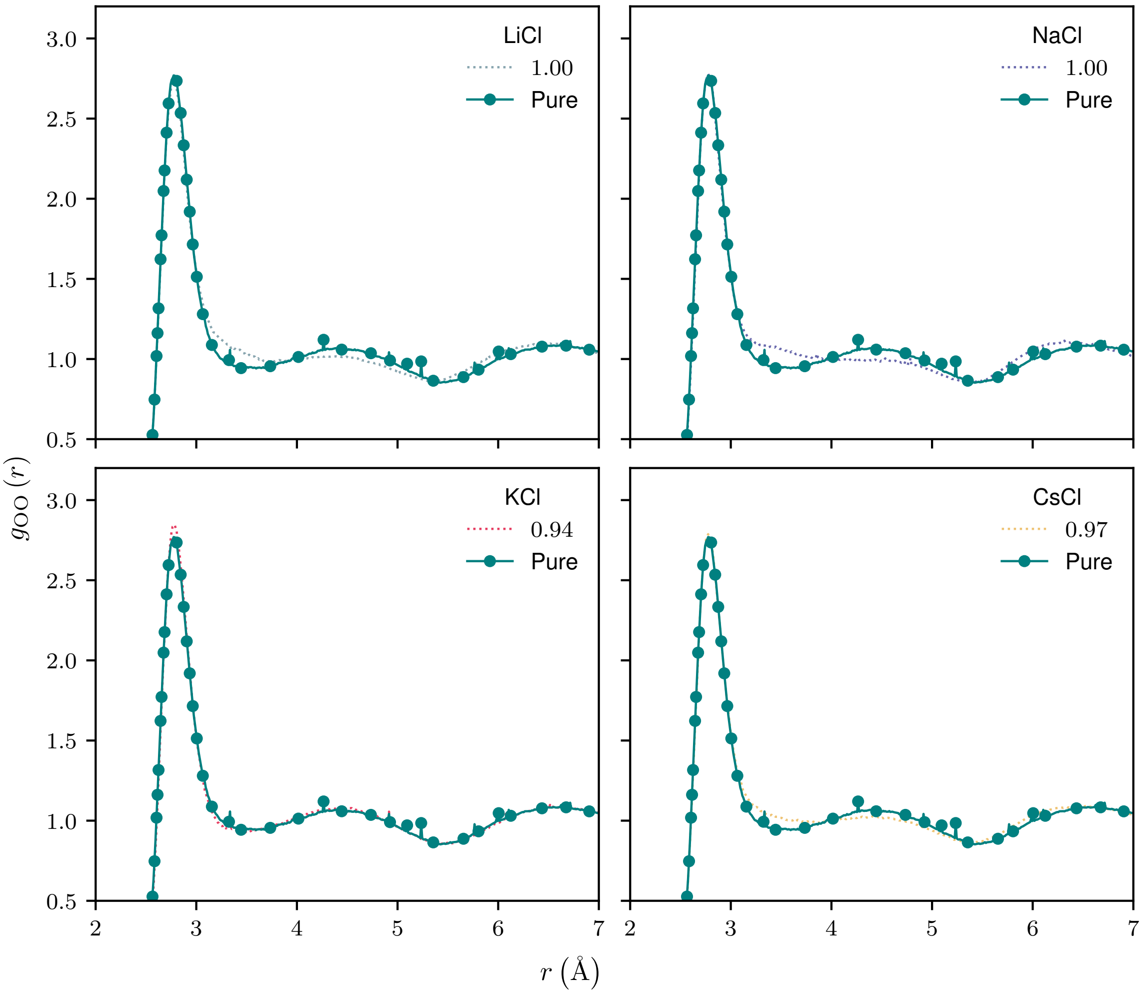

Figure S1. BLYP-D3 Oxygen-Oxygen RDF

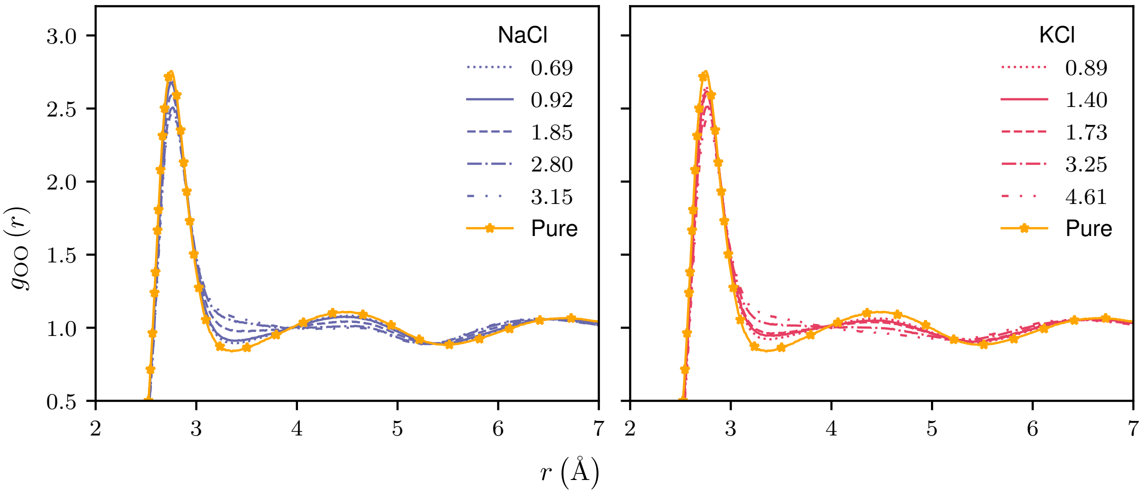

Figure S2. DPMD Oxygen-Oxygen RDF

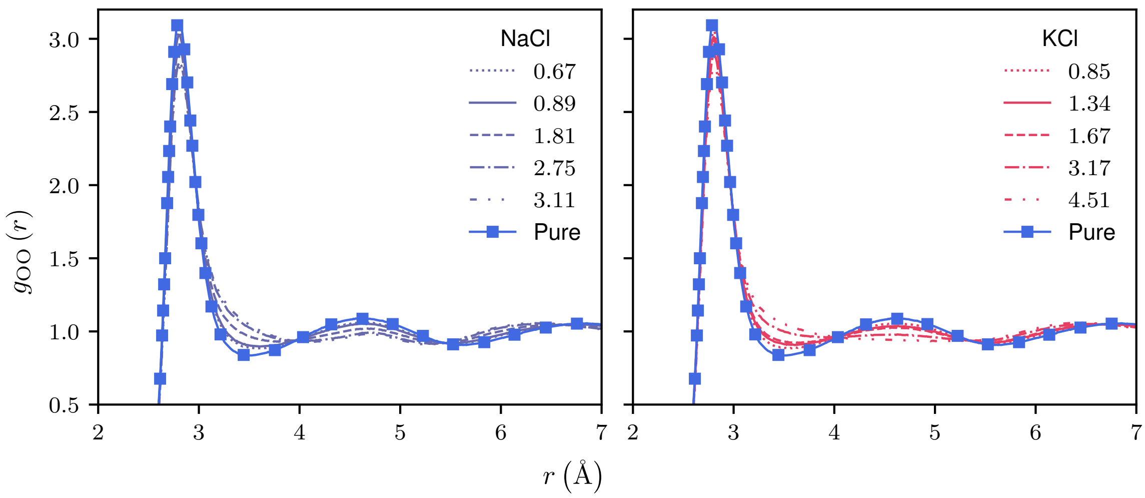

Figure S3. AMOEBA Oxygen-Oxygen RDF

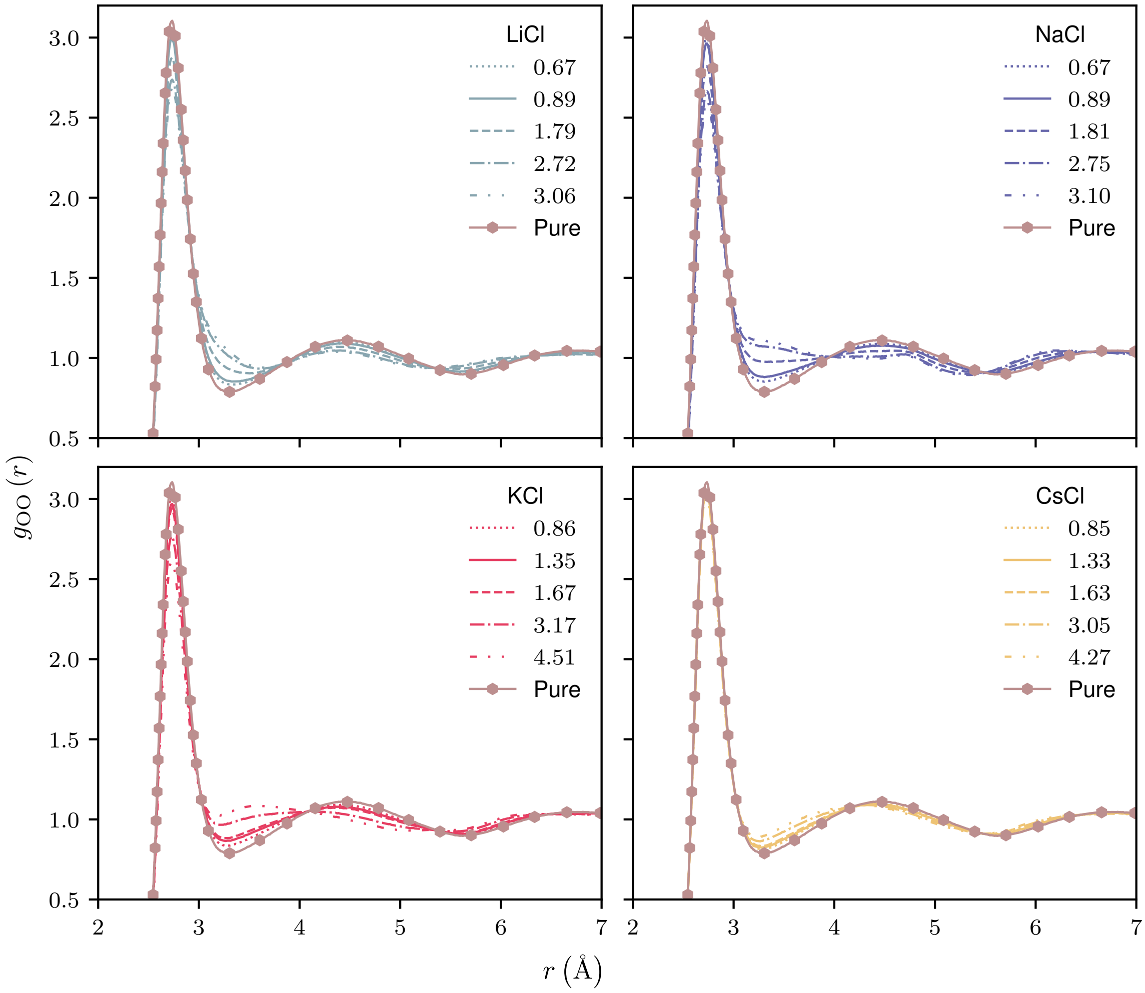

Figure S4. SPC/Fw+JC Oxygen-Oxygen RDF

Pure water

Temperature Shift for SCAN Functional

The SCAN functional, used to train the DPMD model employed in this study, has been subject to controversy regarding its application with temperature shifts ($\Delta T = 30 - 40$K). Adhering to the convention (*45, 46*), we adopted a $\Delta T = 30$K shift for the DPMD model and utilized the resulting data to facilitate comparisons with other force fields whose temperatures were 30K lower. While a rigorous justification for such manipulation falls outside the scope of this work, some rationale can be found in the observation that the 330K relaxation profile (depicted in Figure S6b) exhibits similarities with the 300K profiles obtained from MB-Pol and SPC/Fw water models.

Correlation Time

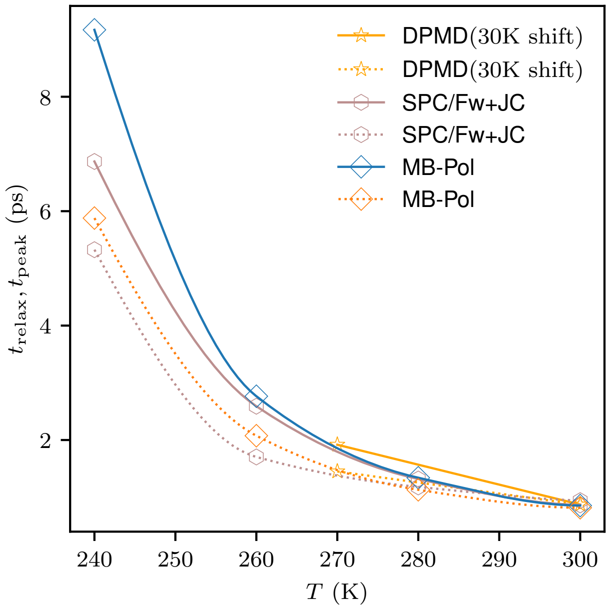

Figure S5. Comparison of t_{peak} and t_{relax} across various temperatures. The dotted line represents t_{peak}, while the solid line corresponds to t_{relax}.

The quantities t_{peak} and t_{relax} are derived from different observables, but they are associated with the same timescale of correlated dynamics. The time t_{peak} corresponds to the value of t that maximizes the four-point correlation function $\chi_4^{SS}(t)$. On the other hand, t_{relax} is the time at which the relaxation function $\langle Q^S(t) \rangle / N_O$ decays to e^{-1} of its initial value. Despite

their different definitions, these two timescales should correspond to each other, as they both characterize the correlated dynamical behavior of the system.

As illustrated in Figure S5, there is indeed a correspondence between $t_{\rm relax}$ and $t_{\rm peak}$. However, a slight decoupling can be observed, likely due to statistical challenges arising from the slowdown of dynamics, which necessitates longer sampling times, particularly for the susceptibility calculations. To address this issue and ensure accurate scaling, we have used $t_{\rm relax}$ instead of $t_{\rm peak}$ for the correlation-scaled diffusivity.

Temperature Dependence of Water Dynamics

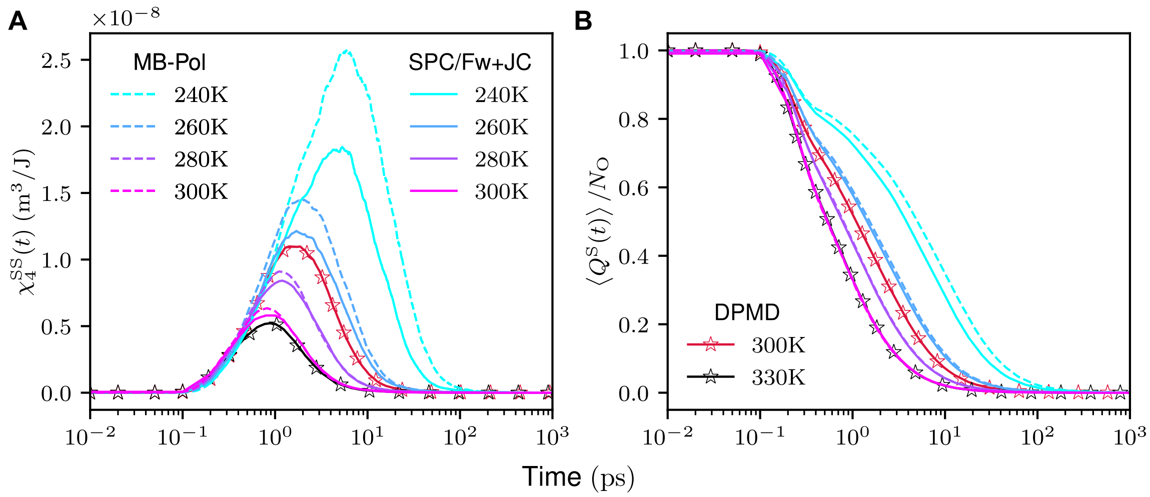

Figure S6. Temperature dependence of four-point susceptibility and relaxation functions for pure water across different force fields. (a) Self-part of four-point susceptibility, $\chi_4^{SS}(t)$, for DPMD, MB-Pol, and SPC/Fw+JC. (b) Corresponding relaxation function, $Q^S(t)/N_O$, profiles

As water is supercooled below the freezing point, the correlation times quantified by $t_{\rm peak}$ from $\chi_4^{SS}(t)$ (Figure S6a) and $t_{\rm relax}$ from $\langle Q^S(t)\rangle/N_O$ (Figure S6b) increase drastically. Concurrently, the correlation lengths, represented by the growing $\chi_4^{SS}(t = t_{\rm peak})$ values (Figure S6a), also expand. This behavior implies that force fields incapable of capturing the enhanced dynamics of supercooled water effectively model a "colder" system upon salt addition, exhibiting increased correlation lengths and times akin to deeply supercooled conditions. Conversely, the DPMD model can produce both higher and lower effective temperatures depending on whether chaotropic or kosmotropic salts are present at sufficiently high concentrations. However, at lower

salt levels, the non-monotonic variations in the peak heights and correlation times of $\chi_4^{\mathrm{SS}}(t)$ suggest more intricate phenomena beyond a simple effective temperature interpretation.

Notably, while the MB-Pol and SPC/Fw+JC force fields demonstrate minor discrepancies in relaxation (Figure S6b) and four-point susceptibility (Figure S6a) near ambient conditions, these deviations amplify substantially in deeper supercooled regimes. This observation highlights the inherent wide spectrum among force field representations of correlated water dynamics, stemming not only from salt effects but also from temperature dependencies.

DPMD Simulation Movie

Movie S1. The representative DPMD simulation movie of CsCl saline solution. This movie demonstrates the stability of DPMD simulations, even when no explicit topological information (e.g., bonds, angles, and dihedrals) is not included.

Time-evolution behavior of $Q(t)$

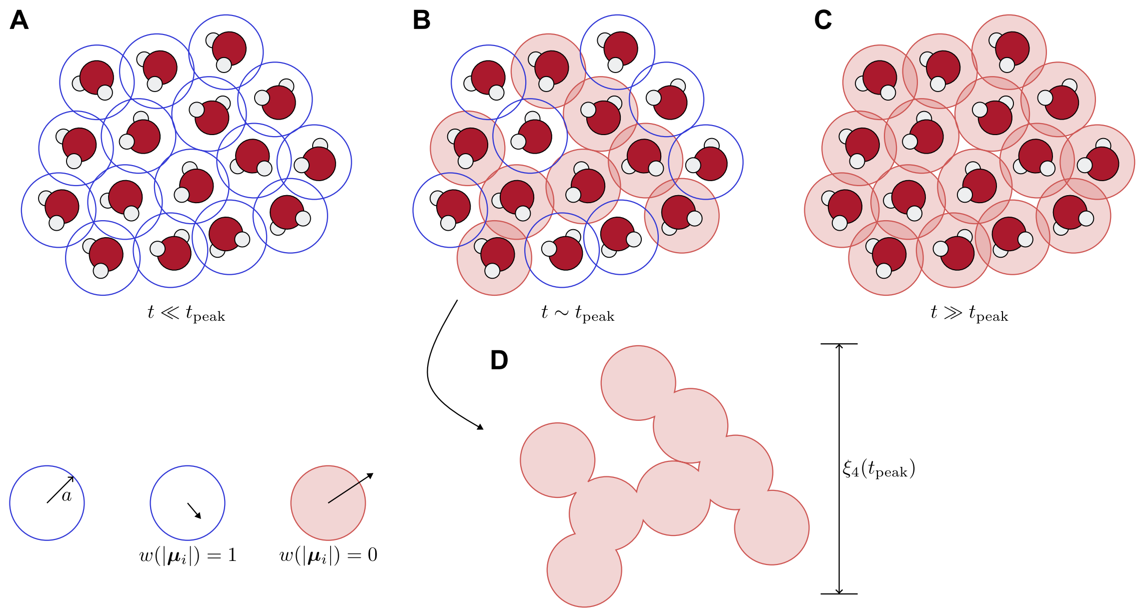

Figure S7. Schematic of time evolution of the system resulting in the decay of $Q(t)$. The blue circle surrounding water molecule denotes the range $w(r) = 1$ is maintained. On the other hand, the shaded red circle denotes the $w(r) = 0$. Note that displacement vector is not drawn for clarity while displacement should be assumed from the position where water molecule is drawn

When the measurement starts ($t = 0$) or only a small amount of time elapses after the start ($t \ll t_{\text{peak}}$), there is no displacement of water molecules over the length $a = 1$Å. Thus, $\langle Q(t) \rangle$ becomes N_O, where N_O is the number of water molecules, as shown in Figure S7A. As time evolves, a fraction of water molecules diffuse from their initial positions, and $Q(t)$ starts to decay. After a characteristic time, t_{relax} or t_{peak}, $\langle Q(t) \rangle / N_O = e^{-1}$ is reached, as shown in Figure S7B. The four-point susceptibility, $\chi_4(t = t_{\text{peak}})$, characterizes the largest fluctuation in the measurement of $Q(t)$ with different choices of initial time, meaning collective excitation of $w(r)$ from 1 to 0. $\chi_4(t = t_{\text{peak}})$ is related to the number of such events (correlated movement of water molecules) and can be related to ξ_4, the length scale of the correlated region as shown in Figure S7D, with an exponent ζ ranging from 2 to 4. Finally, all water molecules diffuse from their original positions, and $\langle Q(t) \rangle$ approaches zero for $t \gg t_{\text{peak}}$ as shown in Figure S7C.

Temperature dependence of diffusivity and correlation-scaled diffusivity

The diffusivity (Figure S8) and correlation-scaled diffusivity (Figure S9) data of SPC/Fw+JC are presented. The ratio of water diffusivity in saline solution to that in pure water (D/D_0) remains constant across the tested temperature range. For correlation-scaled diffusivity, slightly more fluctuation is observed compared to the diffusivity, owing to the susceptibility calculation requiring more statistical data. However, these fluctuations are not substantial enough to cause a qualitative crossover between kosmotrope and chaotrope behavior. Consequently, the choice of temperature has limited influence on the overall results. It should be noted that the melting temperature of SCAN functional water (299K, 311K) (*93, 94*) is higher than the experimental value (273K). Therefore, caution should be taken when considering temperatures below 330K.

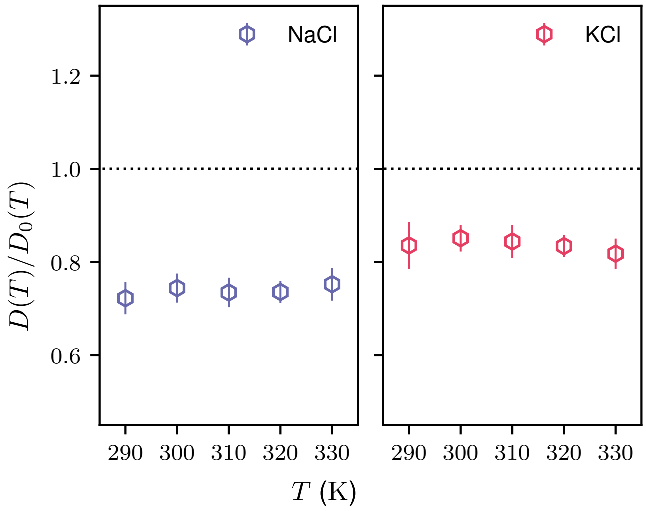

Figure S8. The temperature dependence of D/D_0 in SPC/Fw+JC. The number of water molecules is fixed to 4096 while 128 ion pairs of NaCl and KCl are added to respective saline solution. While thermal expansion makes saline solution with slightly different concentrations, the mean concentration is about 1.7M.

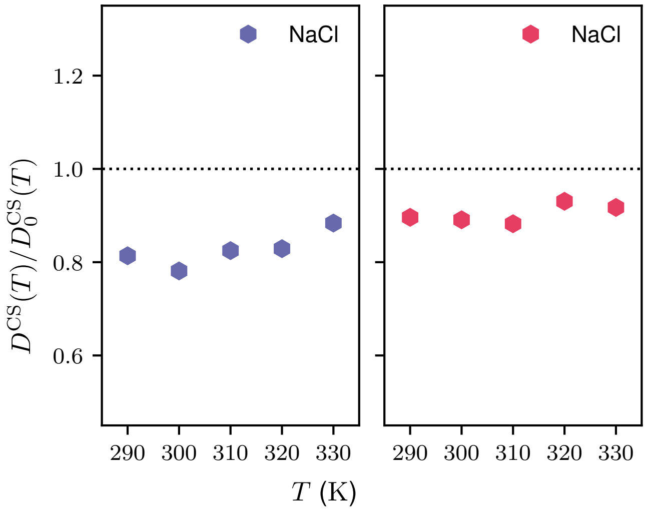

Figure S9. The temperature dependence of D^{CS}/D_0^{CS} in SPC/Fw+JC. The number of water molecules is fixed to 4096 while 128 ion pairs of NaCl and KCl are added to respective saline solution. While thermal expansion makes saline solution with slightly different concentrations, the mean concentration is about 1.7M.

Implementation

In this section, part of the CP2K or LAMMPS script required for defining interactions for each different saline solution is provided. Note that some of the force fields cannot work in a stand-alone manner, and relevant parameter files should be obtained from the repository explained. Relevant data file and the rest of settings such as timestep or integrator should be provided to run the simulation.

DFT (BLYP-D3)

Water

```
&FORCE_EVAL
    &DFT
        &XC
          &XC_FUNCTIONAL BLYP
          &END XC_FUNCTIONAL
          &vdW_POTENTIAL
            DISPERSION_FUNCTIONAL PAIR_POTENTIAL
            &PAIR_POTENTIAL TYPE DFTD3 ### or DFTD3(BJ)
                    CALCULATE_C9_TERM .TRUE.
                    PARAMETER_FILE_NAME dftd3.dat
                    REFERENCE_FUNCTIONAL BLYP
            &END PAIR_POTENTIAL
           &END vdW_POTENTIAL
        &END XC
    &END DFT

    &SUBSYS
        &KIND H
          BASIS_SET DZVP-MOLOPT-SR-GTH
          POTENTIAL GTH-BLYP-q1
        &END KIND
        &KIND O
          BASIS_SET DZVP-MOLOPT-SR-GTH
          POTENTIAL GTH-BLYP-q6
```

```
    &END KIND
  &END SUBSYS
&END FORCE_EVAL
```

LiCl

```
&FORCE_EVAL
  &DFT
    &XC
      &XC_FUNCTIONAL BLYP
      &END XC_FUNCTIONAL
      &vdW_POTENTIAL
        DISPERSION_FUNCTIONAL PAIR_POTENTIAL
        &PAIR_POTENTIAL TYPE DFTD3 ### or DFTD3(BJ)
                CALCULATE_C9_TERM .TRUE.
                PARAMETER_FILE_NAME dftd3.dat
                REFERENCE_FUNCTIONAL BLYP
        &END PAIR_POTENTIAL
       &END vdW_POTENTIAL
    &END XC
  &END DFT

  &SUBSYS
    &KIND H
      BASIS_SET DZVP-MOLOPT-SR-GTH
      POTENTIAL GTH-BLYP-q1
    &END KIND
```

```
        &KIND O
          BASIS_SET DZVP-MOLOPT-SR-GTH
          POTENTIAL GTH-BLYP-q6
        &END KIND
        &KIND Li
          BASIS_SET DZVP-MOLOPT-SR-GTH
          POTENTIAL GTH-BLYP-q3
        &END KIND
        &KIND Cl
          BASIS_SET DZVP-MOLOPT-SR-GTH
          POTENTIAL GTH-BLYP-q7
        &END KIND
    &END SUBSYS
&END FORCE_EVAL
```

NaCl

```
&FORCE_EVAL
    &DFT
        &XC
          &XC_FUNCTIONAL BLYP
          &END XC_FUNCTIONAL
          &vdW_POTENTIAL
            DISPERSION_FUNCTIONAL PAIR_POTENTIAL
            &PAIR_POTENTIAL TYPE DFTD3 ### or DFTD3(BJ)
                    CALCULATE_C9_TERM .TRUE.
                    PARAMETER_FILE_NAME dftd3.dat
```

```
                    REFERENCE_FUNCTIONAL BLYP
            &END PAIR_POTENTIAL
          &END vdW_POTENTIAL
        &END XC
    &END DFT

    &SUBSYS
        &KIND H
          BASIS_SET DZVP-MOLOPT-SR-GTH
          POTENTIAL GTH-BLYP-q1
        &END KIND
        &KIND O
          BASIS_SET DZVP-MOLOPT-SR-GTH
          POTENTIAL GTH-BLYP-q6
        &END KIND
        &KIND Na
          BASIS_SET DZVP-MOLOPT-SR-GTH
          POTENTIAL GTH-BLYP-q9
        &END KIND
        &KIND Cl
          BASIS_SET DZVP-MOLOPT-SR-GTH
          POTENTIAL GTH-BLYP-q7
        &END KIND
    &END SUBSYS
&END FORCE_EVAL
```

KCl

```
&FORCE_EVAL
    &DFT
        &XC
          &XC_FUNCTIONAL BLYP
          &END XC_FUNCTIONAL
          &vdW_POTENTIAL
            DISPERSION_FUNCTIONAL PAIR_POTENTIAL
            &PAIR_POTENTIAL TYPE DFTD3 ### or DFTD3(BJ)
                    CALCULATE_C9_TERM .TRUE.
                    PARAMETER_FILE_NAME dftd3.dat
                    REFERENCE_FUNCTIONAL BLYP
            &END PAIR_POTENTIAL
           &END vdW_POTENTIAL
        &END XC
    &END DFT

    &SUBSYS
        &KIND H
          BASIS_SET DZVP-MOLOPT-SR-GTH
          POTENTIAL GTH-BLYP-q1
        &END KIND
        &KIND O
          BASIS_SET DZVP-MOLOPT-SR-GTH
          POTENTIAL GTH-BLYP-q6
        &END KIND
```

```
    &KIND K
      BASIS_SET DZVP-MOLOPT-SR-GTH
      POTENTIAL GTH-BLYP-q9
    &END KIND
    &KIND Cl
      BASIS_SET DZVP-MOLOPT-SR-GTH
      POTENTIAL GTH-BLYP-q7
    &END KIND
  &END SUBSYS
&END FORCE_EVAL
```

CsCl

```
&FORCE_EVAL
  &DFT
    &XC
      &XC_FUNCTIONAL BLYP
      &END XC_FUNCTIONAL
      &vdW_POTENTIAL
        DISPERSION_FUNCTIONAL PAIR_POTENTIAL
        &PAIR_POTENTIAL TYPE DFTD3 ### or DFTD3(BJ)
                CALCULATE_C9_TERM .TRUE.
                PARAMETER_FILE_NAME dftd3.dat
                REFERENCE_FUNCTIONAL BLYP
        &END PAIR_POTENTIAL
       &END vdW_POTENTIAL
    &END XC
```

```
    &END DFT

    &SUBSYS
        &KIND H
          BASIS_SET DZVP-MOLOPT-SR-GTH
          POTENTIAL GTH-BLYP-q1
        &END KIND
        &KIND O
          BASIS_SET DZVP-MOLOPT-SR-GTH
          POTENTIAL GTH-BLYP-q6
        &END KIND
        &KIND Cs
          BASIS_SET DZVP-MOLOPT-SR-GTH
          POTENTIAL GTH-BLYP-q9
        &END KIND
        &KIND Cl
          BASIS_SET DZVP-MOLOPT-SR-GTH
          POTENTIAL GTH-BLYP-q7
        &END KIND
    &END SUBSYS
&END FORCE_EVAL
```

SPC/Fw+JC

In the following LAMMPS script, type $1-4$ corresponds to oxygen, hydrogen, cation, and anion, respectively.

Water

```
units           metal
pair_style      lj/cut/coul/long 14.0
bond_style      harmonic
angle_style     harmonic
dihedral_style  none
improper_style  none

pair_coeff      1 1     0.006739880631 3.165492
pair_coeff      2 2     0.00 0.00

pair_modify     mix arithmetic tail yes

bond_coeff      1 22.964812 1.012
angle_coeff     1 1.64567 113.24
```

LiCl

```
units           metal
pair_style      lj/cut/coul/long 14.0
bond_style      harmonic
angle_style     harmonic
dihedral_style  none
improper_style  none
```

```
pair_coeff      1 1     0.006739880631 3.165492
pair_coeff      2 2     0.00 0.00
pair_coeff      3 3     0.0146023 1.4094
pair_coeff      4 4     0.0005544 4.83045

pair_modify     mix arithmetic tail yes

bond_coeff      1 22.964812 1.012
angle_coeff     1 1.64567 113.24
```

NaCl

```
units           metal
pair_style      lj/cut/coul/long 14.0
bond_style      harmonic
angle_style     harmonic
dihedral_style  none
improper_style  none

pair_coeff      1 1     0.006739880631 3.165492
pair_coeff      2 2     0.00 0.00
pair_coeff      3 3     0.01529213 2.15954
pair_coeff      4 4     0.0005544 4.83045

pair_modify     mix arithmetic tail yes
```

```
bond_coeff      1 22.964812 1.012
angle_coeff     1 1.64567 113.24
```

KCl

```
units           metal
pair_style      lj/cut/coul/long 14.0
bond_style      harmonic
angle_style     harmonic
dihedral_style  none
improper_style  none

pair_coeff      1 1     0.006739880631 3.165492
pair_coeff      2 2     0.00 0.00
pair_coeff      3 3     0.018634 2.8384
pair_coeff      4 4     0.0005544 4.83045

pair_modify     mix arithmetic tail yes

bond_coeff      1 22.964812 1.012
angle_coeff     1 1.64567 113.24
```

CsCl

```
units           metal
```

```
pair_style      lj/cut/coul/long 14.0
bond_style      harmonic
angle_style     harmonic
dihedral_style  none
improper_style  none

pair_coeff      1 1     0.006739880631 3.165492
pair_coeff      2 2     0.00 0.00
pair_coeff      3 3     0.0038966 3.60101
pair_coeff      4 4     0.0005544 4.83045

pair_modify     mix arithmetic tail yes

bond_coeff      1 22.964812 1.012
angle_coeff     1 1.64567 113.24
```

AMOEBA

The use of AMOEBA in LAMMPS requires parameter (`.prm`) and key (`.key`) files. TINKER `.xyz` data can be converted to LAMMPS format using the `.prm` file and the `tinker2lmp.py` script found in the `lammps/tools/` directory of the LAMMPS root. Each chemical type defined in the TINKER data file is automatically converted and there is no need for additional parameter modifications for different saline solutions. It's important to ensure that the corresponding chemical species are properly defined and accurately converted. The parameter file (`amoebabio18.prm`) used in this study is available in the official TINKER GitHub params directory.

For all chemicals

```
units           real
atom_style      amoeba
bond_style      class2
angle_style     amoeba
dihedral_style  none

fix             amtype all property/atom i_amtype ghost yes
fix             extra all property/atom i_amgroup d_redID d_pval ghost yes
fix             extra2 all property/atom i_polaxe d2_xyzaxis 3

read_data       mixture.data fix amtype NULL "Tinker Types"

pair_style      amoeba
pair_coeff      * * amoebabio18.prm mixture.key
special_bonds   lj/coul 0.5 0.5 0.5 one/five yes
```

In the following `.key` file, `RELEVANT_CELL_SIZE` should be replaced with the box size.

---

mixture.key

```
!! DATA : TODAY'S DATE    UNITS: real
parameters ./amoebabio18.prm
digits 10
a-axis RELEVANT_CELL_SIZE
```

```
ewald
ewald-alpha 0.4
pewald-alpha 0.5
pme-grid 64 64 64
neighbor-list
vdw-cutoff 10.0
polar-eps 1e-4
polar-iter 200
usolve-diag 1.0
```

MB-Pol

MB-Pol requires `mbx.json` which controls the relevant parameters for MB-Pol. Note that this is not included in LAMMPS as a default package at the time of writing. The source code or plugin should be obtained from MBX GitHub repository and properly compiled or activated.

Water

```
units           real
atom_style      full
boundary        p p p

pair_style      mbx 9.0
pair_modify     mix arithmetic

bond_style      none
angle_style     none
dihedral_style  none
```

```
improper_style  none

pair_coeff      * * 0.0 0.0

neighbor        2.0 bin
neigh_modify    every 1 delay 0 check yes

fix             2 all mbx 1 h2o 1 2 3 1 2 2 json mbx.json
```

NINE_COMPONENT_BOX_VECTOR_LIST should be replaced with 9-component box vector.

---

mbx.json

```
{
   "Note" : "This is a cofiguration file",
   "MBX" : {
       "box" : NINE_COMPONENT_BOX_VECTOR_LIST,
       "twobody_cutoff"   : 9.0,
       "threebody_cutoff" : 4.5,
       "max_n_eval_1b"    : 500,
       "max_n_eval_2b"    : 500,
       "max_n_eval_3b"    : 500,
       "dipole_tolerance" : 1E-8,
       "dipole_max_it"    : 100,
       "dipole_method"    : "aspc",
       "alpha_ewald_elec" : 0.60,
       "grid_density_elec" : 2.5,
```

```
        "spline_order_elec" : 6,
        "alpha_ewald_disp" : 0.60,
        "grid_density_disp" : 2.5,
        "spline_order_disp" : 6,
        "ttm_pairs" : [],
        "ignore_2b_poly" : [],
        "ignore_3b_poly" : []
    }
}
```

DPMD

To use DPMD in LAMMPS, LAMMPS must be either compiled with DPMD source or have the plugin enabled. The installation guide in the official DPMD documentation provides further details on this process. The DPMD model file (`.pb`) is available for download from the figshare page associated with Zhang et al.'s publication (*81*) "Dissolving salt is not equivalent to applying a pressure on water". Depending on the version of the installed DPMD package, the model might require conversion for model compatibility. The model compatibility section in the DPMD official documentation contains information about this process.

For simulation of each system, a data file with chemical types corresponding to the model file should be prepared. Type $1-4$ corresponds to oxygen, hydrogen, cation, and anion, respectively.

Water

```
units           metal
atom_style      full
read_data       init.final nocoeff
```

```
neighbor        2.0 bin
neigh_modify    every 1 delay 0 check yes

pair_style deepmd NaCl.pb # alternatively, KCl.pb can be used
pair_coeff * *
```

NaCl

```
units           metal
atom_style      full
read_data       init.final nocoeff
neighbor        2.0 bin
neigh_modify    every 1 delay 0 check yes

pair_style deepmd NaCl.pb
pair_coeff * *
```

KCl

```
units           metal
atom_style      full
read_data       init.final nocoeff
neighbor        2.0 bin
neigh_modify    every 1 delay 0 check yes
```

```
pair_style deepmd KCl.pb

pair_coeff * *
```

REFERENCES AND NOTES

1. J. L. Finney, The structure of water: A historical perspective. *J. Chem. Phys.* **160**, 060901 (2024).

2. P. Gallo, K. Amann-Winkel, C. A. Angell, M. A. Anisimov, F. Caupin, C. Chakravarty, E. Lascaris, T. Loerting, A. Z. Panagiotopoulos, J. Russo, J. A. Sellberg, H. E. Stanley, H. Tanaka, C. Vega, L. Xu, L. G. M. Pettersson, Water: A tale of two liquids. *Chem. Rev.* **116**, 7463–7500 (2016).

3. D. T. Limmer, D. Chandler, The putative liquid-liquid transition is a liquid-solid transition in atomistic models of water. *J. Chem. Phys.* **135**, 134503 (2011).

4. V. Holten, D. T. Limmer, V. Molinero, M. A. Anisimov, Nature of the anomalies in the supercooled liquid state of the mW model of water. *J. Chem. Phys.* **138**, 174501 (2013).

5. K. J. Müller, H. G. Hertz, A parameter as an indicator for water-water association in solutions of strong electrolytes. *J. Phys. Chem.* **100**, 1256–1265 (1996).

6. A. W. Omta, M. F. Kropman, S. Woutersen, H. J. Bakker, Negligible effect of ions on the hydrogen-bond structure in liquid water. *Science* **301**, 347–349 (2003).

7. C. Vega, J. L. Abascal, M. Conde, J. Aragones, What ice can teach us about water interactions: A critical comparison of the performance of different water models. *Faraday Discuss.* **141**, 251–276 (2009).

8. I. Shvab, R. J. Sadus, Atomistic water models: Aqueous thermodynamic properties from ambient to supercritical conditions. *Fluid Phase Equilib.* **407**, 7–30 (2016).

9. A. V. Onufriev, S. Izadi, Water models for biomolecular simulations. *Wiley Interdiscip. Rev. Comput. Mol. Sci.* **8**, e1347 (2018).

10. I. N. Tsimpanogiannis, O. A. Moultos, L. F. M. Franco, M. B. de M. Spera, M. Erdős, I. G. Economou, Self-diffusion coefficient of bulk and confined water: A critical review of classical molecular simulation studies. *Mol. Simul.* **45**, 425–453 (2019).

11. J. S. Kim, Z. Wu, A. R. Morrow, A. Yethiraj, A. Yethiraj, Self-diffusion and viscosity in electrolyte solutions. *J. Phys. Chem. B* **116**, 12007–12013 (2012).

12. Y. Ding, A. A. Hassanali, M. Parrinello, Anomalous water diffusion in salt solutions. *Proc. Natl. Acad. Sci. U.S.A.* **111**, 3310–3315 (2014).

13. Y. Yao, Y. Kanai, M. L. Berkowitz, Role of charge transfer in water diffusivity in aqueous ionic solutions. *J. Phys. Chem. Lett.* **5**, 2711–2716 (2014).

14. Y. Yao, M. L. Berkowitz, Y. Kanai, Communication: Modeling of concentration dependent water diffusivity in ionic solutions: Role of intermolecular charge transfer. *J. Chem. Phys.* **143**, 241101 (2015).

15. M. Andreev, A. Chremos, J. de Pablo, J. F. Douglas, Coarse-grained model of the dynamics of electrolyte solutions. *J. Phys. Chem. B* **121**, 8195–8202 (2017).

16. M. Andreev, J. J. de Pablo, A. Chremos, J. F. Douglas, Influence of ion solvation on the properties of electrolyte solutions. *J. Phys. Chem. B* **122**, 4029–4034 (2018).

17. J. Yoo, A. Aksimentiev, Improved parametrization of Li^+, Na^+, K^+, and Mg^{2+} ions for all-atom molecular dynamics simulations of nucleic acid systems. *J. Phys. Chem. Lett.* **3**, 45–50 (2012).

18. J. Yoo, A. Aksimentiev, New tricks for old dogs: Improving the accuracy of biomolecular force fields by pair-specific corrections to non-bonded interactions. *Phys. Chem. Chem. Phys.* **20**, 8432–8449 (2018).

19. J. Behler, M. Parrinello, Generalized neural-network representation of high-dimensional potential-energy surfaces. *Phys. Rev. Lett.* **98**, 146401 (2007).

20. L. Zhang, J. Han, H. Wang, R. Car, W. E, Deep potential molecular dynamics: A scalable model with the accuracy of quantum mechanics. *Phys. Rev. Lett.* **120**, 143001 (2018).

21. K. Lee, D. Yoo, W. Jeong, S. Han, SIMPLE-NN: An efficient package for training and executing neural-network interatomic potentials. *Comput. Phys. Commun.* **242**, 95–103 (2019).

22. O. T. Unke, S. Chmiela, H. E. Sauceda, M. Gastegger, I. Poltavsky, K. T. Schütt, A. Tkatchenko, K. R. Müller, Machine learning force fields. *Chem. Rev.* **121**, 10142–10186 (2021).

23. Y. Park, J. Kim, S. Hwang, S. Han, Scalable parallel algorithm for graph neural network interatomic potentials in molecular dynamics simulations. *J. Chem. Theory Comput.* **20**, 4857–4868 (2024).

24. I. Poltavsky, A. Tkatchenko, Machine learning force fields: Recent advances and remaining challenges. *J. Phys. Chem. Lett.* **12**, 6551–6564 (2021).

25. X. Fu, Z. Wu, W. Wang, T. Xie, S. Keten, R. Gomez-Bombarelli, T. Jaakkola, Forces are not enough: Benchmark and critical evaluation for machine learning force fields with molecular simulations. arXiv:2210.07237 (2022).

26. G. Kim, B. Na, G. Kim, H. Cho, S. Kang, H. S. Lee, S. Choi, H. Kim, S. Lee, Y. Kim, "Benchmark of machine learning force fields for semiconductor simulations: Datasets, metrics, and comparative analysis" in *NIPS '23: Proceedings of the 37th International Conference on Neural Information Processing Systems* (ACM, 2024), vol. 36, pp. 51434–51476.

27. A. Rodriguez, S. Lam, M. Hu, Thermodynamic and transport properties of lif and flibe molten salts with deep learning potentials. *ACS Appl. Mater. Interfaces* **13**, 55367–55379 (2021).

28. C. Malosso, L. Zhang, R. Car, S. Baroni, D. Tisi, Viscosity in water from first-principles and deep-neural-network simulations. *npj Comput. Mater.* **8**, 139 (2022).

29. T. E. Gartner III, P. M. Piaggi, R. Car, A. Z. Panagiotopoulos, P. G. Debenedetti, Liquid-liquid transition in water from first principles. *Phys. Rev. Lett.* **129**, 255702 (2022).

30. C. Zhang, M. Puligheddu, L. Zhang, R. Car, G. Galli, Thermal conductivity of water at extreme conditions. arXiv:2305.05047 (2023).

31. M. C. Andrade, R. Car, A. Selloni, Probing the self-ionization of liquid water with ab initio deep potential molecular dynamics. *Proc. Natl. Acad. Sci. U.S.A.* **120**, e2302468120 (2023).

32. N. V. S. Avula, M. L. Klein, S. Balasubramanian, Understanding the anomalous diffusion of water in aqueous electrolytes using machine learned potentials. *J. Phys. Chem. Lett.* **14**, 9500–9507 (2023).

33. A. Z. Panagiotopoulos, S. Yue, Dynamics of aqueous electrolyte solutions: Challenges for simulations. *J. Phys. Chem. B* **127**, 430–437 (2023).

34. J. H. Ryu, J. W. Yu, T. J. Yoon, W. B. Lee, Understanding the dielectric relaxation of liquid water using neural network potential and classical pairwise potential. *J. Mol. Liq.* **397**, 124054 (2024).

35. Y. Wu, H. L. Tepper, G. A. Voth, Flexible simple point-charge water model with improved liquid-state properties. *J. Chem. Phys.* **124**, 024503 (2006).

36. I. S. Joung, T. E. Cheatham III, Determination of alkali and halide monovalent ion parameters for use in explicitly solvated biomolecular simulations. *J. Phys. Chem. B* **112**, 9020–9041 (2008).

37. P. Ren, J. W. Ponder, Polarizable atomic multipole water model for molecular mechanics simulation. *J. Phys. Chem. B* **107**, 5933–5947 (2003).

38. R. A. Corrigan, G. Qi, A. C. Thiel, J. R. Lynn, B. D. Walker, T. L. Casavant, L. Lagardere, J. P. Piquemal, J. W. Ponder, P. Ren, M. J. Schnieders, Implicit solvents for the polarizable atomic multipole amoeba force field. *J. Chem. Theory Comput.* **17**, 2323–2341 (2021).

39. A. D. Becke, Density-functional exchange-energy approximation with correct asymptotic behavior. *Phys. Rev. A* **38**, 3098–3100 (1988).

40. C. Lee, W. Yang, R. G. Parr, Development of the colle-salvetti correlation-energy formula into a functional of the electron density. *Phys. Rev. B* **37**, 785–789 (1988).

41. S. Grimme, J. Antony, S. Ehrlich, H. Krieg, A consistent and accurate ab initio parametrization of density functional dispersion correction (DFT-D) for the 94 elements H-Pu. *J. Chem. Phys.* **132**, 154104 (2010).

42. V. Babin, C. Leforestier, F. Paesani, Development of a "first principles" water potential with flexible monomers: Dimer potential energy surface, VRT spectrum, and second virial coefficient. *J. Chem. Theory Comput.* **9**, 5395–5403 (2013).

43. V. Babin, G. R. Medders, F. Paesani, Development of a "first principles" water potential with flexible monomers. II: Trimer potential energy surface, third virial coefficient, and small clusters. *J. Chem. Theory Comput.* **10**, 1599–1607 (2014).

44. G. R. Medders, V. Babin, F. Paesani, Development of a "first-principles" water potential with flexible monomers. III. liquid phase properties. *J. Chem. Theory Comput.* **10**, 2906–2910 (2014).

45. M. Chen, H. Y. Ko, R. C. Remsing, M. F. Calegari Andrade, B. Santra, Z. Sun, A. Selloni, R. Car, M. L. Klein, J. P. Perdew, X. Wu, Ab initio theory and modeling of water. *Proc. Natl. Acad. Sci. U.S.A.* **114**, 10846–10851 (2017).

46. A. Bankura, A. Karmakar, V. Carnevale, A. Chandra, M. L. Klein, Structure, dynamics, and spectral diffusion of water from first-principles molecular dynamics. *J. Phys. Chem. C* **118**, 29401–29411 (2014).

47. G. Stirnemann, E. Wernersson, P. Jungwirth, D. Laage, Mechanisms of acceleration and retardation of water dynamics by ions. *J. Am. Chem. Soc.* **135**, 11824–11831 (2013).

48. S. Shimizu, N. Matubayasi, Ion hydration: Linking self-diffusion and reorientational motion to water structure. *Phys. Chem. Chem. Phys.* **20**, 5909–5917 (2018).

49. E. Pluhařová, G. Stirnemann, D. Laage, On water reorientation dynamics in cation hydration shells. *J. Mol. Liq.* **363**, 119886 (2022).

50. R. Shi, A. J. Cooper, H. Tanaka, Impact of hierarchical water dipole orderings on the dynamics of aqueous salt solutions. *Nat. Commun.* **14**, 4616 (2023).

51. A. Offei-Danso, U. N. Morzan, A. Rodriguez, A. Hassanali, A. Jelic, The collective burst mechanism of angular jumps in liquid water. *Nat. Commun.* **14**, 1345 (2023).

52. P. Banerjee, B. Bagchi, Role of local order in anomalous ion diffusion: Interrogation through tetrahedral entropy of aqueous solvation shells. *J. Chem. Phys.* **153**, 154505 (2020).

53. R. A. X. Persson, V. Pattni, A. Singh, S. M. Kast, M. Heyden, Signatures of solvation thermodynamics in spectra of intermolecular vibrations. *J. Chem. Theory Comput.* **13**, 4467–4481 (2017).

54. S. Kumar, B. Bagchi, Anomalous concentration dependence of viscosity: Hidden role of cross-correlations in aqueous electrolyte solutions. *J. Phys. Chem. B* **127**, 11031–11044 (2023).

55. K. J. Tielrooij, N. Garcia-Araez, M. Bonn, H. J. Bakker, Cooperativity in ion hydration. *Science* **328**, 1006–1009 (2010).

56. D. A. Turton, J. Hunger, G. Hefter, R. Buchner, K. Wynne, Glasslike behavior in aqueous electrolyte solutions. *J. Chem. Phys.* **128**, 161102 (2008).

57. E. B. Moore, V. Molinero, Growing correlation length in supercooled water. *J. Chem. Phys.* **130**, 244505 (2009).

58. T. Kawasaki, K. Kim, Identifying time scales for violation/preservation of stokes-einstein relation in supercooled water. *Sci. Adv.* **3**, e1700399 (2017).

59. R. Shi, J. Russo, H. Tanaka, Origin of the emergent fragile-to-strong transition in supercooled water. *Proc. Natl. Acad. Sci. U.S.A.* **115**, 9444–9449 (2018).

60. S. C. Glotzer, V. N. Novikov, T. B. Schrøder, Time-dependent, four-point density correlation function description of dynamical heterogeneity and decoupling in supercooled liquids. *J. Chem. Phys.* **112**, 509–512 (2000).

61. N. Lačević, F. W. Starr, T. B. Schrøder, S. C. Glotzer, Spatially heterogeneous dynamics investigated via a time-dependent four-point density correlation function. *J. Chem. Phys.* **119**, 7372–7387 (2003).

62. M. Vogel, S. C. Glotzer, Temperature dependence of spatially heterogeneous dynamics in a model of viscous silica. *Phys. Rev. E Stat. Nonlin. Soft Matter Phys.* **70**, 061504 (2004).

63. A. S. Keys, A. R. Abate, S. C. Glotzer, D. J. Durian, Measurement of growing dynamical length scales and prediction of the jamming transition in a granular material. *Nat. Phys.* **3**, 260–264 (2007).

64. S. K. Reddy, S. C. Straight, P. Bajaj, C. Huy Pham, M. Riera, D. R. Moberg, M. A. Morales, C. Knight, A. W. Götz, F. Paesani, On the accuracy of the MB-pol many-body potential for water: Interaction energies, vibrational frequencies, and classical thermodynamic and dynamical properties from clusters to liquid water and ice. *J. Chem. Phys.* **145**, 194504 (2016).

65. B. B. Bizzarro, C. K. Egan, F. Paesani, Nature of halide–water interactions: Insights from many-body representations and density functional theory. *J. Chem. Theory Comput.* **15**, 2983–2995 (2019).

66. C. K. Egan, B. B. Bizzarro, M. Riera, F. Paesani, Nature of alkali ion–water interactions: Insights from many-body representations and density functional theory. II. *J. Chem. Theory Comput.* **16**, 3055–3072 (2020).

67. L. Berthier, G. Biroli, J. P. Bouchaud, L. Cipelletti, D. E. Masri, D. L'Hôte, F. Ladieu, M. Pierno, Direct experimental evidence of a growing length scale accompanying the glass transition. *Science* **310**, 1797–1800 (2005).

68. R. S. Stein, H. C. Andersen, Scaling analysis of dynamic heterogeneity in a supercooled lennard-jones liquid. *Phys. Rev. Lett.* **101**, 267802 (2008).

69. E. Flenner, G. Szamel, Dynamic heterogeneity in a glass forming fluid: Susceptibility, structure factor, and correlation length. *Phys. Rev. Lett.* **105**, 217801 (2010).

70. L. Berthier, G. Biroli, Theoretical perspective on the glass transition and amorphous materials. *Rev. Mod. Phys.* **83**, 587–645 (2011).

71. G. Brambilla, D. El Masri, M. Pierno, L. Berthier, L. Cipelletti, G. Petekidis, A. B. Schofield, Probing the equilibrium dynamics of colloidal hard spheres above the mode-coupling glass transition. *Phys. Rev. Lett.* **102**, 085703 (2009).

72. T. Narumi, S. V. Franklin, K. W. Desmond, M. Tokuyama, E. R. Weeks, Spatial and temporal dynamical heterogeneities approaching the binary colloidal glass transition. *Soft Matter* **7**, 1472–1482 (2011).

73. M. P. Longinotti, M. A. Carignano, I. Szleifer, H. R. Corti, Anomalies in supercooled NaCl aqueous solutions: A microscopic perspective. *J. Chem. Phys.* **134**, 244510 (2011).

74. P. Garbacz, W. S. Price, ^{1}H NMR diffusion studies of water self-diffusion in supercooled aqueous sodium chloride solutions. *Chem. A Eur. J.* **118**, 3307–3312 (2014).

75. L. Martínez, R. Andrade, E. G. Birgin, J. M. Martínez, Packmol: A package for building initial configurations for molecular dynamics simulations. *J. Comput. Chem.* **30**, 2157–2164 (2009).

76. A. P. Thompson, H. M. Aktulga, R. Berger, D. S. Bolintineanu, W. M. Brown, P. S. Crozier, P. J. in 't Veld, A. Kohlmeyer, S. G. Moore, T. D. Nguyen, R. Shan, M. J. Stevens, J. Tranchida, C. Trott, S. J. Plimpton, LAMMPS - A flexible simulation tool for particle-based materials modeling at the atomic, meso, and continuum scales. *Comput. Phys. Commun.* **271**, 108171 (2022).

77. P. Eastman, J. Swails, J. D. Chodera, R. T. McGibbon, Y. Zhao, K. A. Beauchamp, L. P. Wang, A. C. Simmonett, M. P. Harrigan, C. D. Stern, R. P. Wiewiora, B. R. Brooks, V. S. Pande, Openmm 7: Rapid development of high performance algorithms for molecular dynamics. *PLoS Comput. Biol.* **13**, e1005659 (2017).

78. T. D. Kühne, M. Iannuzzi, M. D. Ben, V. V. Rybkin, P. Seewald, F. Stein, T. Laino, R. Z. Khaliullin, O. Schütt, F. Schiffmann, D. Golze, J. Wilhelm, S. Chulkov, M. H. Bani-

Hashemian, V. Weber, U. Borštnik, M. Taillefumier, A. S. Jakobovits, A. Lazzaro, H. Pabst, T. Müller, R. Schade, M. Guidon, S. Andermatt, N. Holmberg, G. K. Schenter, A. Hehn, A. Bussy, F. Belleflamme, G. Tabacchi, A. Glöß, M. Lass, I. Bethune, C. J. Mundy, C. Plessl, M. Watkins, J. V. Vondele, M. Krack, J. Hutter, CP2K: An electronic structure and molecular dynamics software package - Quickstep: Efficient and accurate electronic structure calculations. *J. Chem. Phys.* **152**, 194103 (2020).

79. M. Holz, S. R. Heil, A. Sacco, Temperature-dependent self-diffusion coefficients of water and six selected molecular liquids for calibration in accurate 1H NMR PFG measurements. *Phys. Chem. Chem. Phys.* **2**, 4740–4742 (2000).

80. L. B. Skinner, C. Huang, D. Schlesinger, L. G. M. Pettersson, A. Nilsson, C. J. Benmore, Benchmark oxygen-oxygen pair-distribution function of ambient water from x-ray diffraction measurements with a wide Q-range. *J. Chem. Phys.* **138**, 074506 (2013).

81. C. Zhang, S. Yue, A. Z. Panagiotopoulos, M. L. Klein, X. Wu, Dissolving salt is not equivalent to applying a pressure on water. *Nat. Commun.* **13**, 822 (2022).

82. P. Giannozzi, O. Andreussi, T. Brumme, O. Bunau, M. Buongiorno Nardelli, M. Calandra, R. Car, C. Cavazzoni, D. Ceresoli, M. Cococcioni, N. Colonna, I. Carnimeo, A. Dal Corso, S. de Gironcoli, P. Delugas, R. A. DiStasio Jr., A. Ferretti, A. Floris, G. Fratesi, G. Fugallo, R. Gebauer, U. Gerstmann, F. Giustino, T. Gorni, J. Jia, M. Kawamura, H. Y. Ko, A. Kokalj, E. Küçükbenli, M. Lazzeri, M. Marsili, N. Marzari, F. Mauri, N. L. Nguyen, H. V. Nguyen, A. Otero-de-la-Roza, L. Paulatto, S. Poncé, D. Rocca, R. Sabatini, B. Santra, M. Schlipf, A. P. Seitsonen, A. Smogunov, I. Timrov, T. Thonhauser, P. Umari, N. Vast, X. Wu, S. Baroni, Advanced capabilities for materials modelling with quantum espresso. *J. Phys. Condens. Matter* **29**, 465901 (2017).

83. J. Sun, A. Ruzsinszky, J. P. Perdew, Strongly constrained and appropriately normed semilocal density functional. *Phys. Rev. Lett.* **115**, 036402 (2015).

84. D. Hamann, M. Schlüter, C. Chiang, Norm-conserving pseudopotentials. *Phys. Rev. Lett.* **43**, 1494–1497 (1979).

85. D. Vanderbilt, Optimally smooth norm-conserving pseudopotentials. *Phys. Rev. B* **32**, 8412–8415 (1985).

86. D. R. Hamann, Optimized norm-conserving Vanderbilt pseudopotentials. *Phys. Rev. B Condens. Matter Mater. Phys.* **88**, 085117 (2013).

87. H. Wang, L. Zhang, J. Han, W. E, DeePMD-kit: A deep learning package for many-body potential energy representation and molecular dynamics. *Comput. Phys. Commun.* **228**, 178–184 (2018).

88. L. Zhang, H. Wang, M. C. Muniz, A. Z. Panagiotopoulos, R. Car, W. E, A deep potential model with long-range electrostatic interactions. *J. Chem. Phys.* **156**, 124107 (2022).

89. W. M. Haynes, *CRC Handbook of Chemistry and Physics* (CRC Press, 2014).

90. R. Mancinelli, A. Botti, F. Bruni, M. A. Ricci, A. K. Soper, Perturbation of water structure due to monovalent ions in solution. *Phys. Chem. Chem. Phys.* **9**, 2959–2967 (2007).

91. R. Mancinelli, A. Botti, F. Bruni, M. A. Ricci, A. K. Soper, Hydration of sodium, potassium, and chloride ions in solution and the concept of structure maker/breaker. *J. Phys. Chem. B* **111**, 13570–13577 (2007).

92. J. A. Rackers, Z. Wang, C. Lu, M. L. Laury, L. Lagardère, M. J. Schnieders, J. P. Piquemal, P. Ren, J. W. Ponder, Tinker 8: Software tools for molecular design. *J. Chem. Theory Comput.* **14**, 5273–5289 (2018).

93. P. Montero de Hijes, C. Dellago, R. Jinnouchi, G. Kresse, Density isobar of water and melting temperature of ice: Assessing common density functionals. *J. Chem. Phys.* **161**, 131102 (2024).

94. P. M. Piaggi, A. Z. Panagiotopoulos, P. G. Debenedetti, R. Car, Phase equilibrium of water with hexagonal and cubic ice using the SCAN functional. *J. Chem. Theory Comput.* **17**, 3065–3077 (2021).
